# Supplementary material for: Metacognitive efficiency in learned value-based choice
Source: PLoS Comput Biol. 2026 Mar 31;22(3):e1014108. doi: 10.1371/journal.pcbi.1014108 (PMC13102309; doi:10.1371/journal.pcbi.1014108)
Supplement: S1 Text — This supplementary text contains Boxes A and B and Figures A−K. Fig A. Recovery analysis for Forward and Backward models. A) In the Forward model, we simulated the behavior of 100 agents using random values for four parameters: learning-rate (α), inverse temperature (β), lower-bound for confidence (L), and upper-bound for confidence (H). We also randomly selected the trajectory of rewards from those experienced by the subjects. Subsequently, we fit the behavior of these agents to the Forward model to recover the parameters. The original values of each of the four parameters were highly correlated with their corresponding recovered values across all agents. B) The same analysis was conducted for the Backward model, based on the confidence ratings of the agents in A. Again, actual and recovered parameters were significantly correlated. The dots in the plots above indicate the respective estimations for each subject. Fig B. The influence of different fitting methods on recovered parameters The 4 recovered parameters; learning-rate (A), inverse-temperature (B), confidence upper (C) and lower bounds (D), were highly correlated between Forward and Backward models. The above plots display dots that represent the relevant estimations for each subject. Fig C. Forward (blue) and Backward (red) performance under influence of confidence noise. A) We created synthetic agents using empirical choices but synthetic confidence ratings. In these agent, called Hmeta(σ2), we first assigned the highest confidence to correct choices and the lowest to incorrect ones, and then corrupted these values by Gaussian noise with variance σ2 (suitably truncated and quantized). We observed that increasing the level of confidence noise in Hmeta(σ2)’s confidence values led to a decrease in Backward performance and constant Forward performance, as was expected. B) The Backward learning-rate decreased with increasing noise variance. C) The inverse-temperature increased as noise variance increased. D-F) [file pcbi.1014108.s001.pdf]

Supplementary Materials:
Metacognitive Efficiency in Learned Value-based Choice

immediate

S1 Text. Supplementary information.

BoxA: Recovery analysis. The Backward and Forward models share common elements, but differ in their fitting methods. Therefore, it is important to perform parameter recovery for both models and verify the consistency of the recovered parameters between them.

The parameters for both Forward and Backward models were randomly selected from the following ranges: $(0, 1)$ for learning-rate, $(0, 100)$ for inverse-temperature, and $(0, 1)$ for the two confidence bound parameters, H_c and L_c (with the constraint that L_c is smaller than H_c) in the range $[1, 5]$ (For simplicity, we call the parameters of both Forward and Backward the same). We simulated the Forward model to generate primary choices and confidence ratings in a task generated at random from one of the 54 empirical sequence of bandit sequences. Then, we fitted the choices to the Forward model to obtain the recovered learning-rate and inverse-temperature, called recovered choice parameters. Subsequently, to recover the confidence bound parameters, we simulated the model's confidence in its primary choices based on the recovered choice parameters, and minimized the quadratic distance between the primary and the scaled confidence rating. This entire procedure was repeated 100 times, with one simulation and 120 rounds of fitting (with different random initializations) for each sampled set of parameters. We treated the parameters achieved from the best fit as the recovered parameters.

Similar steps were followed for the Backward model. We simulated scaled confidence in the primary choices (explained in the above paragraph). Then, the quadratic distance between scaled and the primary confidences was minimized to recover the Backward parameters.

We applied Pearson correlation to assess the quality of recovery for each parameter in the model. We found that the parameters of the Forward model could be recovered reliably using maximum likelihood to fit choices: α (r = 0.98, p$\ll$ 10e-10), β (r = 0.94, p$\ll$ 10e-10), L (r = 1.00, p$\ll$ 10e-10) and H (r = 1.00, p$\ll$ 10e-10) (Fig AA in S1 Text).

We also fit the Backward model to the scaled confidence reports of the subjects simulated using the Forward model, through minimizing the quadratic distance with Backward confidence ratings. The parameters generating the behavior could also be recovered through this Backwards process: α (r = 1.00, p$\ll$ 10e-10), β (r = 1.00, p$\ll$ 10e-10), L (r = 1.00, p$\ll$ 10e-10) and H (r = 1.00, p$\ll$ 10e-10) (Fig AB in S1 text).

The parameters recovered from the Backward model were more closely correlated with the original generating parameters than were the parameters recovered from the Forward

model (α; Z = -31.76, p≪ 10e-10, β; Z = -14.56, p≪ 10e-10). This is consistent with the observation that each data point used to fit the Backward model is a real-valued probability rather than just a binary choice.

Nevertheless, the parameters recovered from the Forward and Backward models were strongly correlated with each other; α (r = 0.98, p≪ 10e-10), β (r = 0.94, p≪ 10e-10), L_c (r = 1.00, p≪ 10e-10) and H_c (r = 1.00, p≪ 10e-10). Thus, the different fitting methods in Forward and Backward models did not influence the recovered parameters (Fig B in S1 Text).

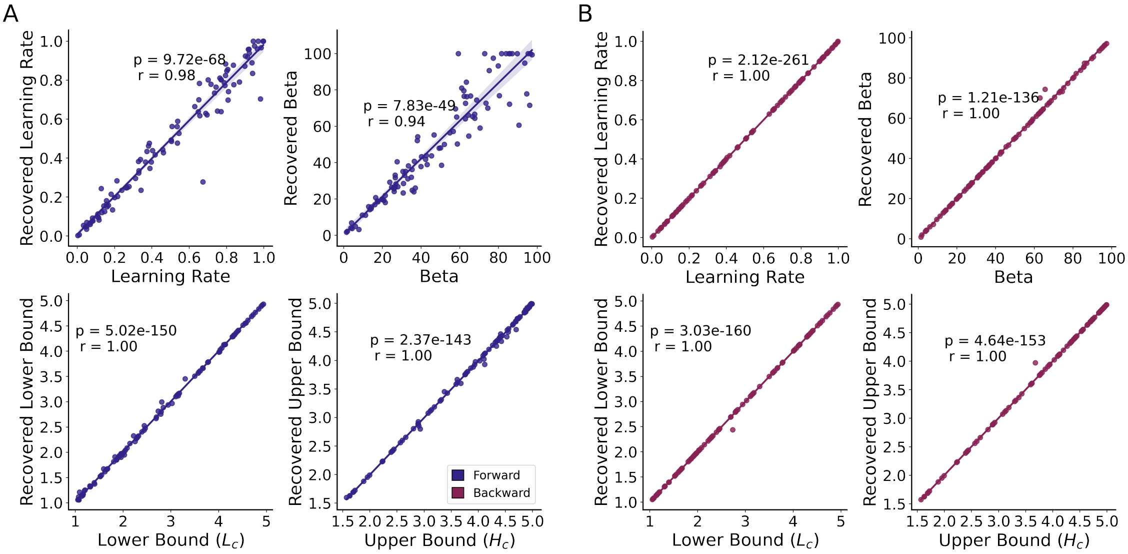

Fig A. Recovery analysis for Forward and Backward models. A) In the Forward model, we simulated the behavior of 100 agents using random values for four parameters: learning-rate (α), inverse temperature (β), lower-bound for confidence (L), and upper-bound for confidence (H). We also randomly selected the trajectory of rewards from those experienced by the subjects. Subsequently, we fitted the behavior of these agents to the Forward model to recover the parameters. The original values of each of the four parameters were highly correlated with their corresponding recovered values across all agents. B) The same analysis was conducted for the Backward model, based on the confidence ratings of the agents in A. Again, actual and recovered parameters were significantly correlated. The dots in the plots above indicate the respective estimations for each subject.

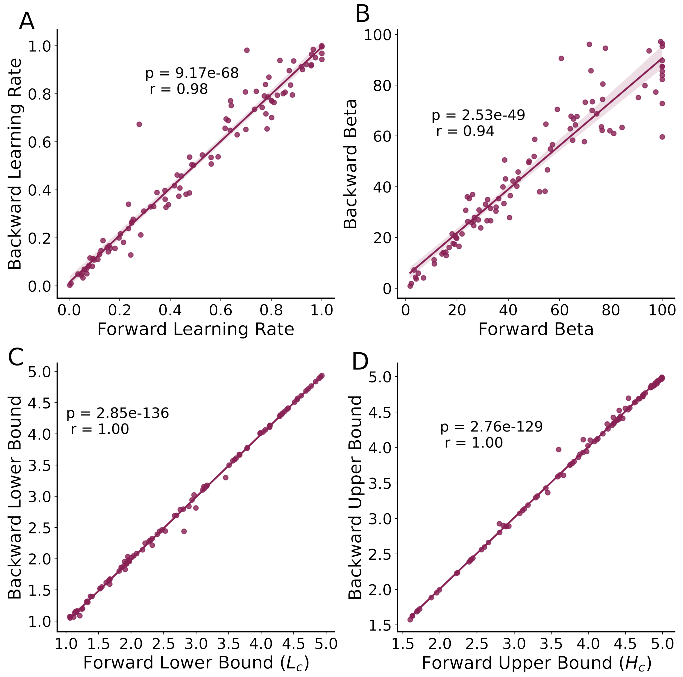

Fig B. The influence of different fitting methods on recovered parameters The 4 recovered parameters; learning-rate (A), inverse-temperature (B), confidence upper (C) and lower bounds (D), were highly correlated between Forward and Backward models. The above plots display dots that represent the relevant estimations for each subject.

Box B: Sensitivity of Backward performance to confidence noise To simulate the behavior of a High-meta agent Hmeta(0), we took the empirical choices a_t^s of participant s, and used our knowledge of the underlying block to endow choices of the bandit that were better on trial t with the highest rating (a value of 5) and of the bandit that was worse, the lowest rating (1). These ratings were then corrupted by noise drawn from a normal distribution with 0 mean and standard deviation sampled at 10 equidistant points within the continuous range $[0, 4]$. If the sampled confidence lay outside our empirical confidence range, $[1, 5]$, it was mapped to the closest bound, either 1 or 5. For each level of standard deviation, the noisy confidence ratings were sampled 100 times. Each sequence of synthetic behavior was fitted 120 times to obtain Forward and Backward parameters, and then the Forward and Backward choices were simulated 100 times. In Fig CA of S1 Text, we show the average (dots) and the standard deviation (error bars) of the Forward and Backward performance across 100 simulations for each standard deviation. The learning-rate and inverse-temperature produced by this procedure were also illustrated in Fig CB and C of S1 Text.

We also examined the impact of different levels of confidence noise σ^2 on the fitted parameters of the Backward model. Higher levels of noise variance were associated with lower learning-rates and higher inverse temperatures (Fig CB and C in S1 Text). This implies that when confidence is more prone to noise, recent experiences carry less weight. Since the model is expressing its (noisy, and so potentially extreme) confidence in the same choices as the subjects, it requires a high inverse-temperature. Thus, the parameters of our Backward model were also sensitive to confidence noise.

We also explored the effect of confidence noise variance on the behavior of a Forward(σ^2) agent —a Forward agent with added confidence noise— by fitting the Backwards model

to its corrupted first-order confidence reports. We found that higher levels of σ^2 were associated with decreased Backward performance (Fig CD in S1 Text). Additionally, we observed a decrease in the learning-rate and an increase in the inverse temperature under the influence of higher levels of noise variance (Fig CE and F in S1 Text). Therefore, our findings regarding the impact of noise variance on Backward performance and parameters were consistent across both types of agents: High-meta(σ^2) and Forward(σ^2).

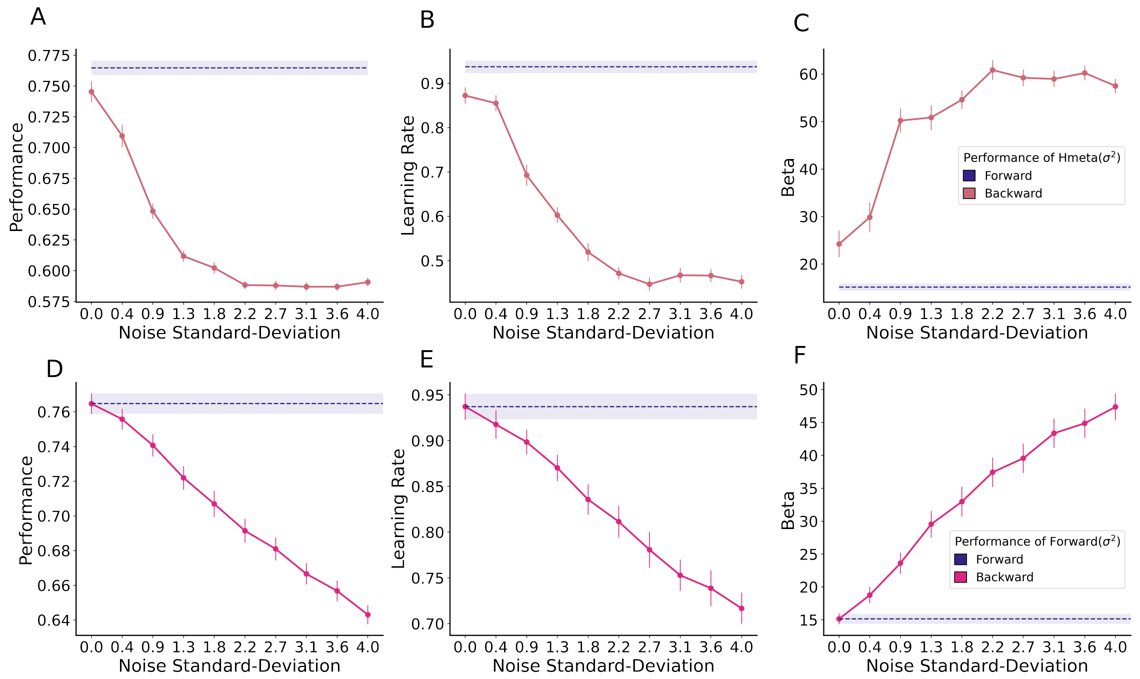

Fig C. Forward (blue) and Backward (red) performance under influence of confidence noise. A) We created synthetic agents using empirical choices but synthetic confidence ratings. In these agent, called Hmeta(σ^2), we first assigned the highest confidence to correct choices and the lowest to incorrect ones, and then corrupted these values by Gaussian noise with variance σ^2 (suitably truncated and quantized). We observed that increasing the level of confidence noise in Hmeta(σ^2)'s confidence values led to a decrease in Backward performance and constant Forward performance, as was expected. B) The Backward learning-rate decreased with increasing noise variance. C) The inverse-temperature increased as noise variance increased. D-F) We repeated the same procedure for simulated choices and confidence ratings coming from the Forward model fit to the same empirical data. Similar to our findings with the Hmeta(σ^2) agent, we observed a decrease in Backward performance under the influence of confidence noise, as expected, while Forward performance remained constant. E) The Backward learning-rate for the Forward(σ^2) agent decreased with higher noise variance. F) The inverse-temperature increased as noise variance increased. The dots in the above plots denote the related estimates for each subject.

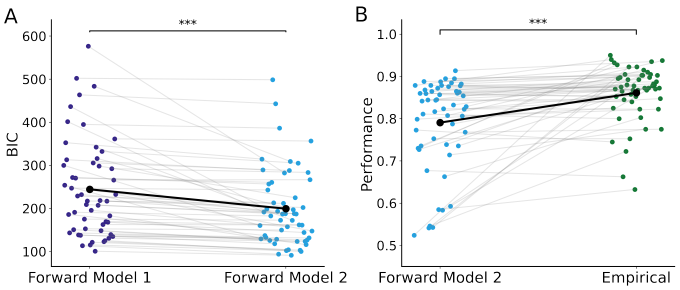

Fig D. Comparison between simpler and more complex Forward model fits. A) The simple two-parameter forward model (1), used throughout most of the text, fits the behavioral data less well than the more complex four-parameter forward model (2). B) Even so, the performance of forward model (2) remained lower than the empirical performance (i.e., participants' performance). The dots in the plots above indicate the corresponding parameter estimates for each subject.

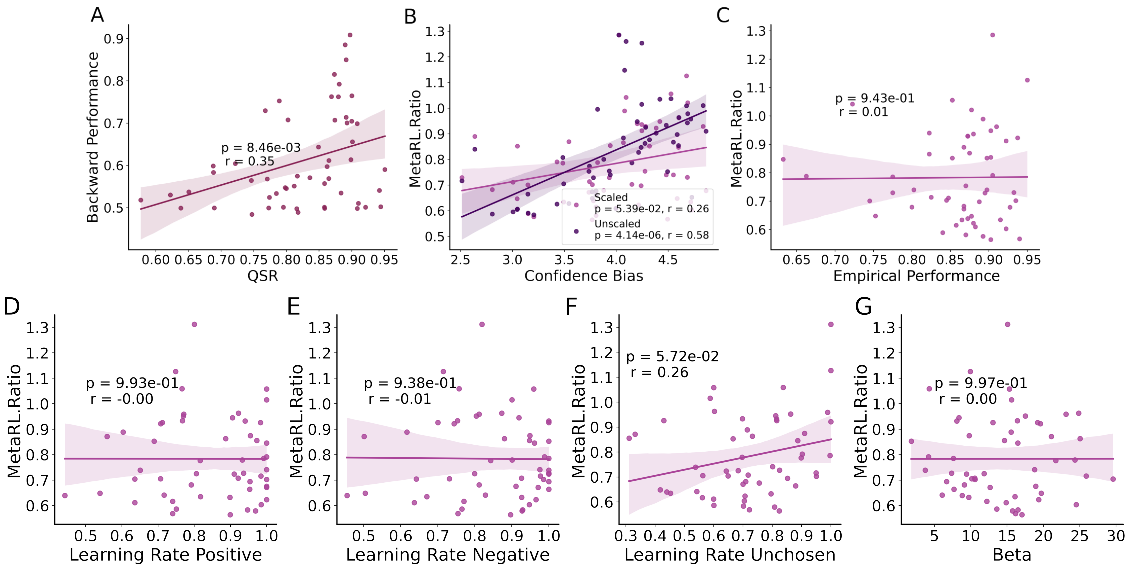

Fig E. Metacognitive measures for more complex Forward model. A) The QSR and Backward performance were positively correlated (r = 0.35, p = 8.46e-03). B) The confidence scaling method decreased the dependency of MetaRL.Ratio on confidence-bias (Z = -3.485, p = 0.0004). C, D, E, F & G) The MetaRL.Ratio was independent of empirical performance (r = 0.01, p = .943), and all Forward parameters (Positive learning-rate; r = 0.00, p = .993, Negative learning-rate; r = -0.01, p = .938, unchosen learning-rate; r = 0.26, p = .057, and β; r = 0.00, p = .997). The dots in the above plots denote the related estimations for each subject.

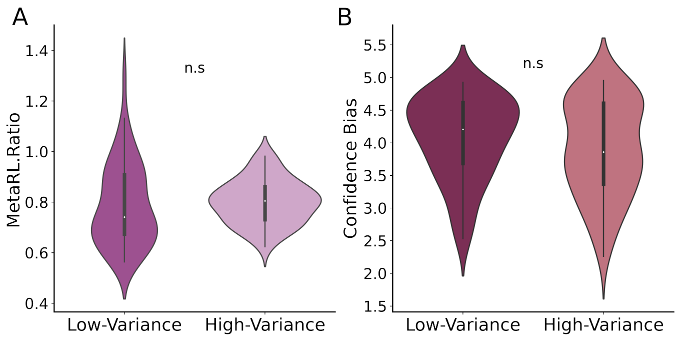

Fig F. MetaRL.Ratio and confidence-bias across levels of difficulty for the more complex Forward model. A) the MetaRL.Ratio was not different between difficulties (W = 641.0, p = .382); B) and nor was the average confidence (W = 540.0, p = .081).

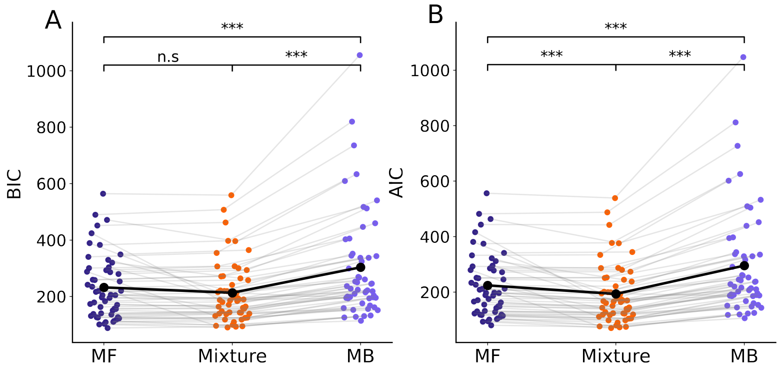

Fig G. Comparison between MF, Mixture, and MB models in low-variance condition of task. A) The goodness of fit, as measured by BIC, was not significantly different between MF and the Mixture model. However, both the Mixture model and MF fit better than the MB model. B) According to AIC, the Mixture model provided a better fit than both MF and MB, while MF fit our empirical data better than MB.

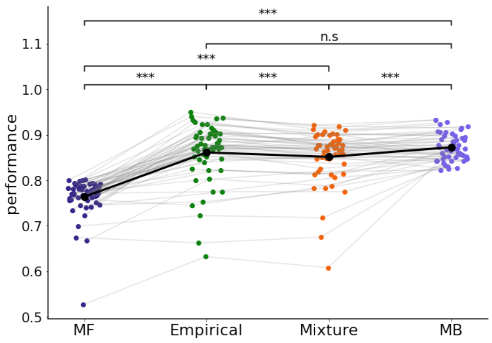

Fig H. Comparison between empirical performance and models performance in the low-variance condition of the task. The performance of MB RL and Mixture models was significantly closer to participants' performance than MF RL (MB RL vs. MF RL: 0.87 vs. 0.76, W = 0, p_{Bonf} = 6.504e-10; Mixture vs. MF RL: 0.85 vs. 0.76, W = 0, p_{Bonf} = 6.504e-10). However, while the Mixture model's performance was significantly different from empirical performance (Mean = 0.85 vs. 0.86, W = 312, p_{Bonf} = 8.4e-04), MB performance was not significantly different from empirical performance (Mean = 0.87 vs. 0.86, W = 686, p_{Bonf} = 1.00). P-values were Bonferroni-corrected across the four tests reported in this paragraph; adjusted p-values are reported.

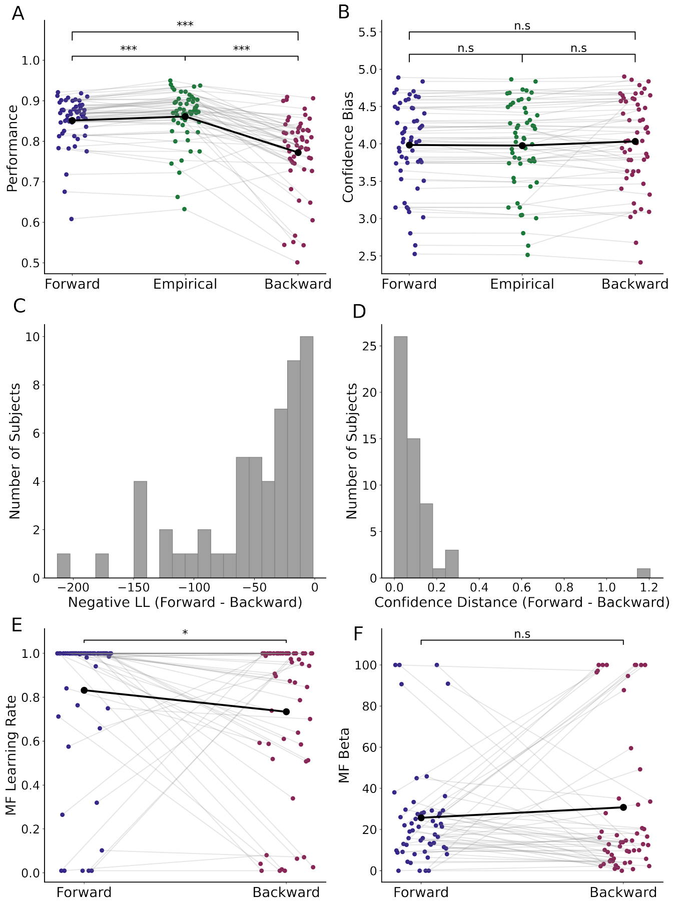

Fig I. Comparison between Forward and Backward Mixture models in choice, confidence and parameters in low-variance condition. A) The performance of the Backward Mixture model was significantly lower than both empirical and Forward performance. Additionally, Forward performance significantly lagged behind empirical performance. B) The confidence-bias levels of the Backward model were not significantly different from the Forward model and empirical data, also there was not a significant difference between the confidence-bias of the Forward model and empirical data. C) The Forward model predicted choices better than the Backward model, as measured by the negative log likelihood. D) The confidence ratings of the Backward model were closer to the empirical data than those of the Forward model. E) The learning-rate was significantly lower in the Backward model compared to the Forward model. F) The inverse-temperature was significantly higher in the Backward model compared to the Forward model. The dots in the plots above represent the corresponding estimations for each subject in the low variance condition of task.

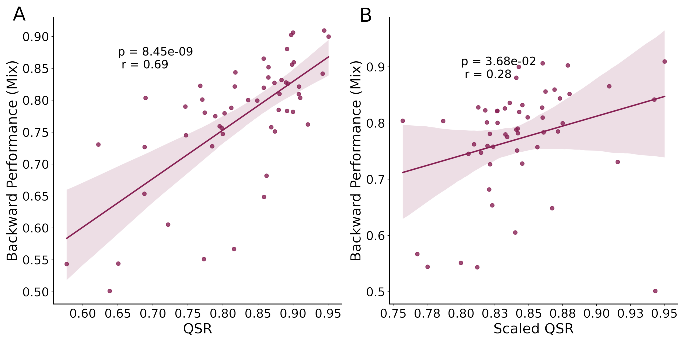

Fig J. Relationship with Quadratic Scoring rule, model-free measure of meta-cognitive sensitivity in low-variance condition of task. A) Backward performance based on the Mixture model was not significantly correlated with QSR. B) Backward performance was also not significantly correlated with scaled-QSR, which determines a linear scaling of empirical confidence values to maximize QSR. The dots in the plots above represent the corresponding estimations for each subject. Each dot in the above plots reflects the associated estimations for the subjects in the low variance condition of task.

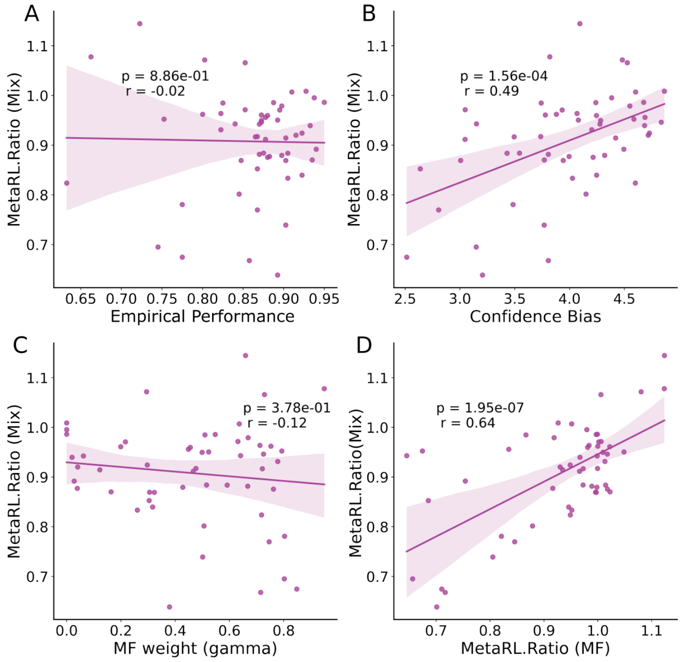

Fig K. Independence of the MetaRL.Ratio, based on Mixture model, from empirical choice parameters in low-variance condition of task. A) The MetaRL.Ratio, our measure of metacognitive efficiency, was independent of empirical performance. B) The correlation between MetaRL.Ratio and confidence-bias decreased after applying the confidence scaling method. C & D) The MetaRL.Ratio was not significantly correlated with the inverse-temperature (C) or the learning-rate (D) of the Forward Mixture model. The above plots display dots that represent the relevant estimations for each subject in the low variance condition of task.
