# Supplementary material for: Metacognitive efficiency in learned value-based choice
Source: PLoS Comput Biol. 2026 Mar 31;22(3):e1014108. doi: 10.1371/journal.pcbi.1014108 (PMC13102309; doi:10.1371/journal.pcbi.1014108)
Supplement: S2 Text — This supplementary text contains Figures A−T. Fig A. Backward learning-rate and auto-correlation in confidence-rates. A&B) Task conditions are shown in order (low-variance, then high-variance). In the Backward model, learning rate was negatively correlated with the autocorrelation of confidence ratings, indicating that lower learning rates were associated with more autocorrelated confidence ratings: low-variance (r=−0.27,p=4.93e−02) and high-variance (r=−0.46,p=4.68e−04). C&D) The same analysis for the Leaky Backward model. In the low-variance condition, the correlation was not significant (r=−0.17,p=2.28e−01), whereas in the high-variance condition, lower learning rates were associated with higher autocorrelation (r=−0.40,p=2.50e−03). Each dot represents one participant. The strength of the correlation did not differ significantly between the Backward and Leaky Backward models in either condition (low-variance: Z = 0.53, p = .59, high-variance: Z = 0.37, p = 0.71). Fig B. Forward and Backward confidence bound parameters. Each dot in the above plots reflects the associated estimations for the subjects. Fig C. The scaled QSR was less dependent on confidence-bias relative to QSR. The dots shown in the above plots correspond to the estimations for each subject. Fig D. Independence of MetaRL.Ratio from Forward parameters in synthetic data. A) The learning-rate was fixed at three levels, the MetaRL.Ratio remained around 1 for 10 equalized distant points of β between 5 and 90. B) The β was fixed at three levels, the MetaRL.Ratio remained around 1 for 10 equalized distant points of β between 0.05 and 1. Fig E. Leaky model of confidence; independence from confidence bias. We also considered an improved, ‘Leaky’ model of confidence which captures autocorrelation in confidence reports by using a weighted average of the ‘true’ confidence on the current trial and the confidence report on the previous trial. A) For some subjects the MetaRL.Ratio from leaky and non-leaky model of [file pcbi.1014108.s002.pdf]

Supplementary Materials: Metacognitive Efficiency in Learned Value-based Choice

immediate

S2 Text. Supplementary information.

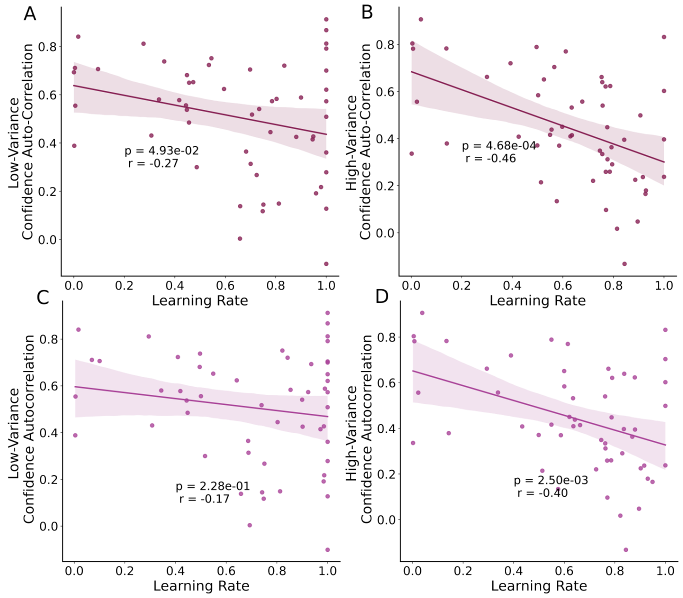

Fig A. Backward learning-rate and auto-correlation in confidence-rates. A&B) Task conditions are shown in order (low-variance, then high-variance). In the Backward model, learning rate was negatively correlated with the autocorrelation of confidence ratings, indicating that lower learning rates were associated with more autocorrelated confidence ratings: low-variance ($r = -0.27, p = 4.93e-02$) and high-variance ($r = -0.46, p = 4.68e-04$). C&D) The same analysis for the Leaky Backward model. In the low-variance condition, the correlation was not significant ($r = -0.17, p = 2.28e-01$), whereas in the high-variance condition, lower learning rates were associated with higher autocorrelation ($r = -0.40, p = 2.50e-03$). Each dot represents one participant. The strength of the correlation did not differ significantly between the Backward and Leaky Backward models in either condition (low-variance: $Z = 0.53, p = .59$, high-variance: $Z = 0.37, p = 0.71$).

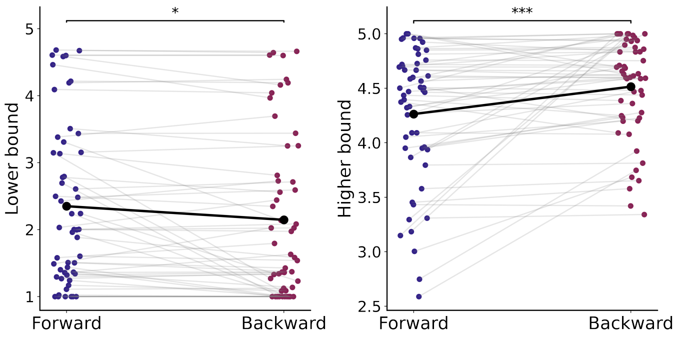

Fig B. Forward and Backward confidence bound parameters. Each dot in the above plots reflects the associated estimations for the subjects.

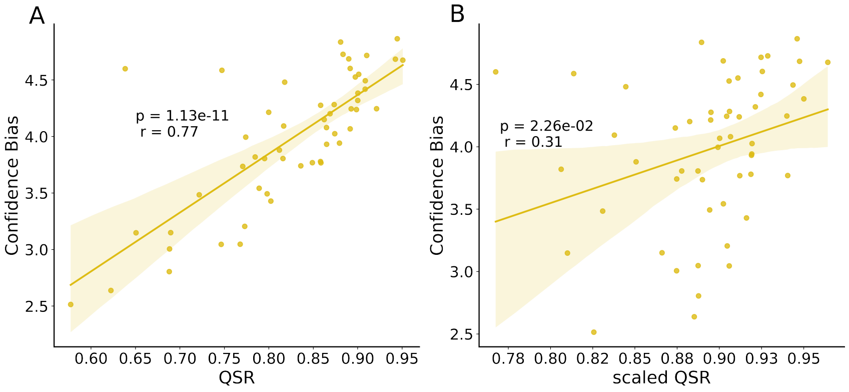

Fig C. The scaled QSR was less dependent on confidence-bias relative to QSR. The dots shown in the above plots correspond to the estimations for each subject.

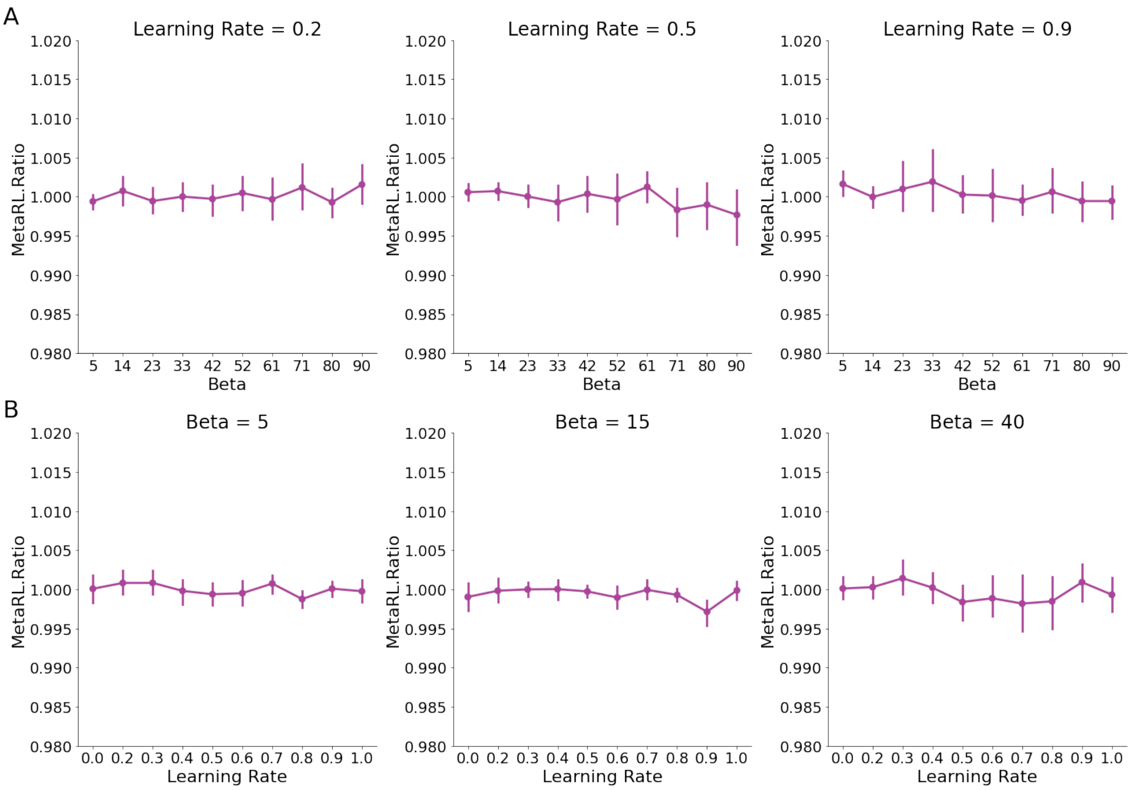

Fig D. Independence of MetaRL.Ratio from Forward parameters in synthetic data. A) The learning-rate was fixed at three levels, the MetaRL.Ratio remained around 1 for 10 equalized distant points of β between 5 and 90. B) The β was fixed at three levels, the MetaRL.Ratio remained around 1 for 10 equalized distant points of β between 0.05 and 1.

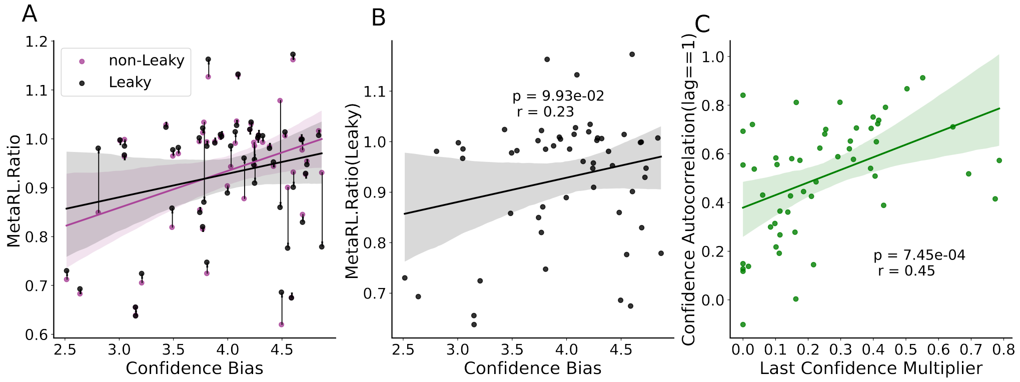

Fig E. Leaky model of confidence; independence from confidence bias. We also considered an improved, 'Leaky' model of confidence which captures autocorrelation in confidence reports by using a weighted average of the 'true' confidence on the current trial and the confidence report on the previous trial. A) For some subjects the MetaRL.Ratio from leaky and non-leaky model of confidence were different from each other. B) When using this leaky model, the MetaRL.Ratio was no longer significantly correlated with the confidence bias. C) The weight accorded to the confidence report on the previous trial, called the 'Last Confidence Multiplier', was positively correlated with the degree of autocorrelation in the confidence reports.

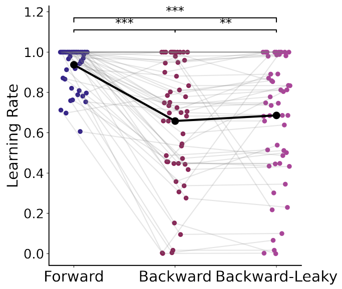

Fig F. Comparison of learning rates between Forward, Backward, and Leaky Backward models. The Backward model had a lower learning rate than the Forward model, and the Leaky Backward model had a higher learning rate than the Backward model while remaining lower than the Forward model.

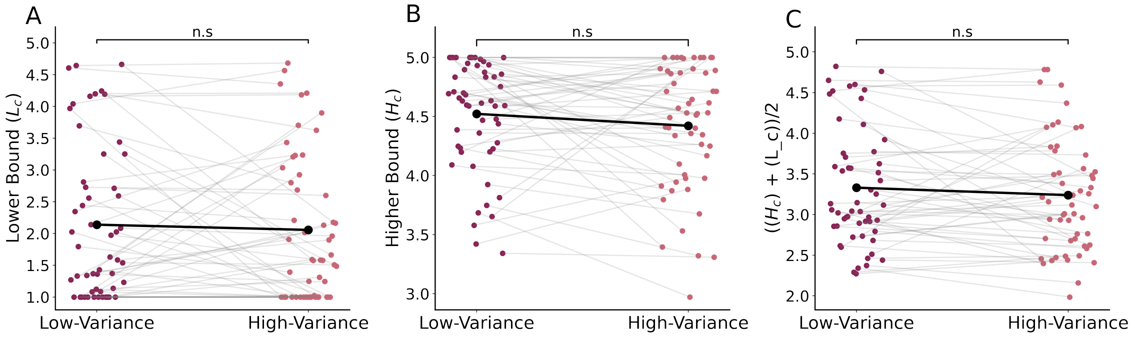

Fig G. Confidence bound parameters were not influenced by task difficulty. A & B) The lower and upper bounds of confidence (L and H) and C) their average were not influenced by task difficulty. The dots in the above plots denote the related estimations for each subject.

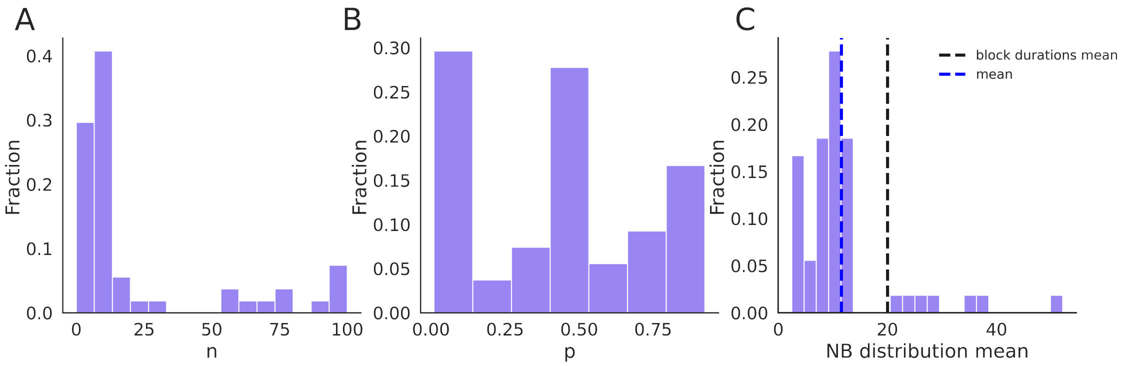

Fig H. Parameters of the Forward MB model. A & B) Fitted n and p parameters of the Negative Binomial distribution for the Forward MB model. C)The corresponding truncated Negative Binomial distribution (capped at 70 trials). The vertical blue dashed line indicates the mean of this distribution, representing the model's reversal trials. In contrast, the vertical black dashed line indicates the mean reversal trial across all subjects and blocks in the empirical data.

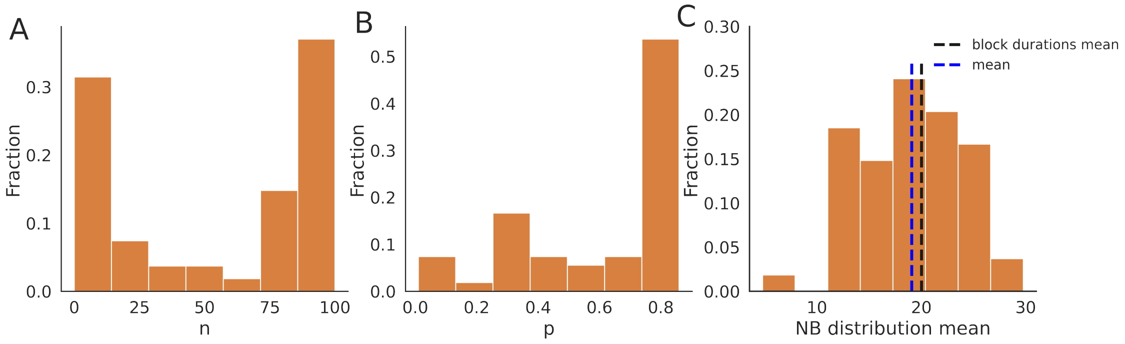

Fig I. Parameters of the Forward Mixture model. A & B) Fitted n and p parameters of the Negative Binomial distribution for the Forward Mixture model. C) The corresponding truncated Negative Binomial distribution (capped at 70 trials). The vertical blue dashed line indicates the mean of this distribution, representing the model's reversal trials. In contrast, the vertical black dashed line indicates the mean reversal trial across all subjects and blocks in the empirical data.

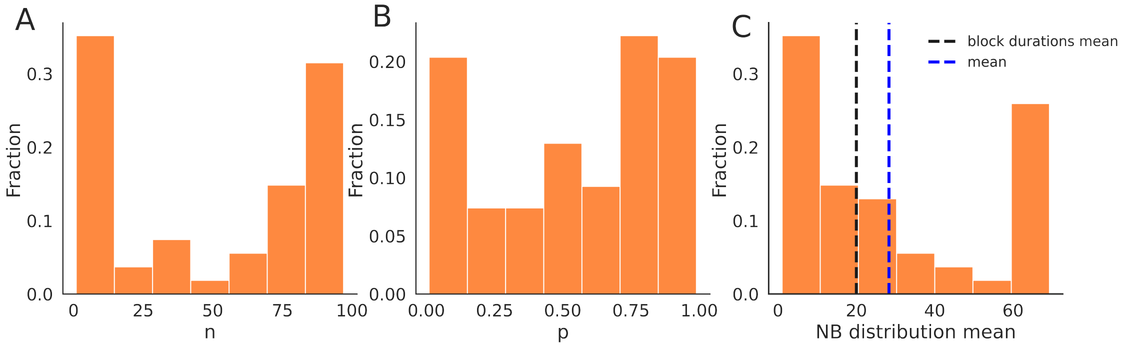

Fig J. Parameters of the Backward Mixture model. A & B) Fitted n and p parameters of the Negative Binomial distribution for the Backward Mixture model. C) The corresponding truncated Negative Binomial distribution (capped at 70 trials). The vertical blue dashed line indicates the mean of this distribution, representing the model's reversal trials. In contrast, the vertical black dashed line indicates the mean reversal trial across all subjects and blocks in the empirical data.

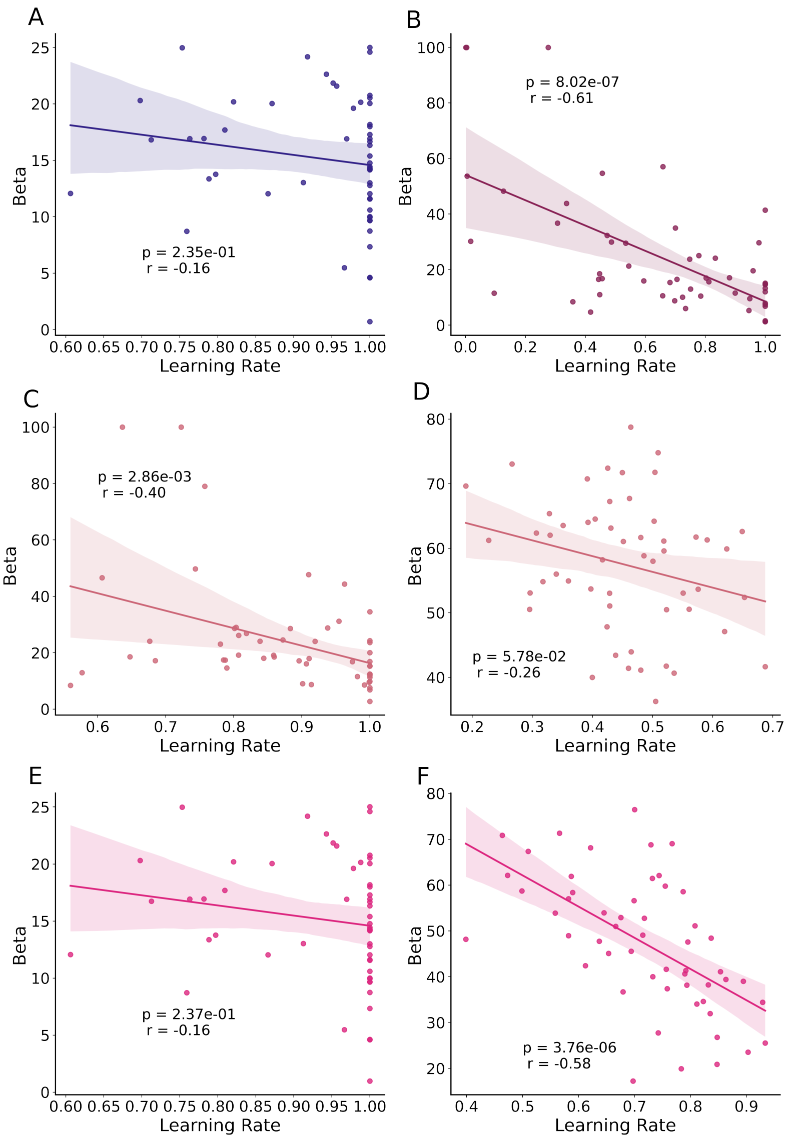

Fig K. Correlation between parameters. A) In the Forward model, the learning rate and inverse temperature were not correlated ($r = -0.16, p = 0.235$). B) However, a negative correlation was observed for these parameters in the Backward model ($r = -0.61, p < 0.001$). C) Specifically, in the H-meta agent, these two Backward parameters exhibited a negative correlation for the noiseless model ($r = -0.40, p = .003$). D) This negative correlation was not evident at a standard deviation of 4 ($r = -0.26, p = .058$). E) For the Forward agent, the correlation in confidence noise with a standard deviation of 0 was not significant ($r = -0.16, p = .237$). F) In contrast, a significant negative correlation was found for confidence noise with a standard deviation of 4 ($r = -0.58, p < 0.001$). The dots in the above plots denote the related estimations for each subject.

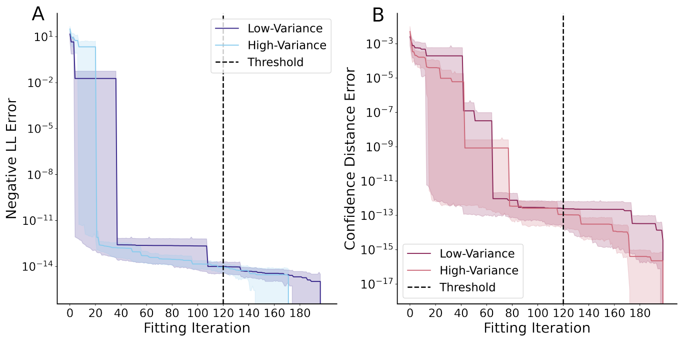

Fig L. Number of required iterations for fitting stability. Mean of (A) Negative Log-Likelihood (LL) and (B) Confidence Distance Error are shown in this plot. The fitting process involves a non-convex optimization problem, where both choice and confidence are fitted to the subject's responses over several iterations, selecting the best-fitting parameters. This plot illustrates the mean absolute differences between the best-fitting parameters at iteration t and the results after 200 iterations. It is evident that the fitting stabilizes for both measures after approximately 120 iterations, which was chosen as the cutoff threshold for generating other plots. The y-axis is on a log scale, and the error bars represent 95% confidence intervals.

Box A: Comparison between three representation of confidence.

There are alternative representations of confidence in computational modeling, such as the $Q-$value of the chosen option or the absolute difference of $Q-$values between two options (**???**). Thus we compared three representations of confidence; Probability of choice (Model1), Q-value for the chosen option (Model2), and the absolute difference between Q-values (Model3). We found that our representation of confidence could predict empirical ratings better than the absolute difference of Q-values ($W = 68.0$, $p = 6.337\text{e-}09$), although it was not significantly different from using the Q-value of the chosen option ($W = 555.0$, $p = .106$) (Fig BA in S2 Text). In addition, the Q-value chosen was closer to empirical confidences than absolute difference of Q-values ($W = 41.0$, $p = 1.54\text{e-}09$). More importantly, our confidence representation resulted in the most sensitive Backward performance to confidence noise; faster decrease in Backward performance as a function of confidence noise compared (the analysis related to Fig C in S1 Text) relative to the other representations (Fig BB in S2 Text).

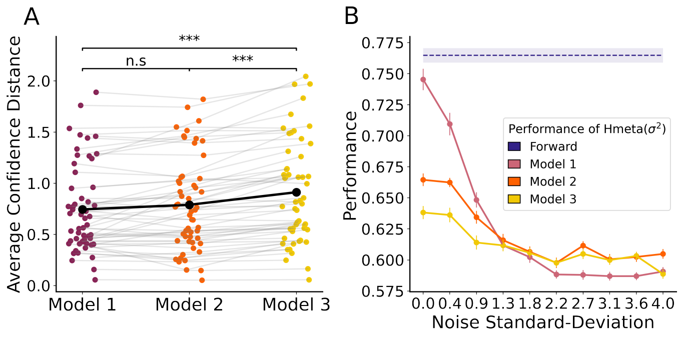

Fig M. Comparison between three representation of confidence A) The Backward model, incorporating the absolute difference between Q-values (Model3), exhibited a significantly superior fit to the empirical data compared to the Backward model that represented confidence using the Probability of choice (Model1). Interestingly, no significant difference was observed between Probability of choice and the Q-value of the chosen option (Model2). Each dot reflects the associated estimations for the subjects. B) The Probability of choice resulted in the most sensitive Backward performance to confidence noise compared with the other representations we tested.

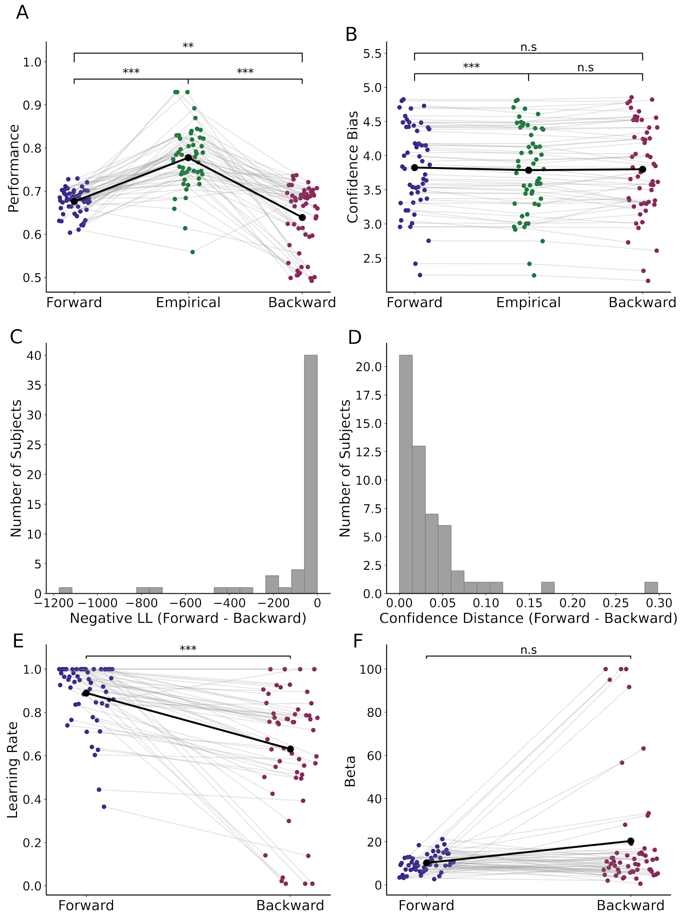

Fig N. Comparison between Forward and Backward models in choice, confidence and parameters in high-variance condition. A) The performance of the Backward model was significantly lower than both empirical and Forward performance. Additionally, Forward performance significantly lagged behind empirical performance. B) The confidence-bias of the Backward model, quantified as empirical average confidence, was not significantly different from that of the Forward model or empirical data, while there was a significant difference between the confidence-bias of the Forward model and empirical data. C) The Forward model predicted choices better than the Backward model, as measured by the negative log likelihood. D) The confidence ratings of the Backward model were closer to the empirical data than those of the Forward model. E) The learning-rate was significantly lower in the Backward model compared to the Forward model. F) The inverse-temperature parameter was not significantly different between two models. The dots in the plots above represent the corresponding estimations for each subject in the high variance condition of task.

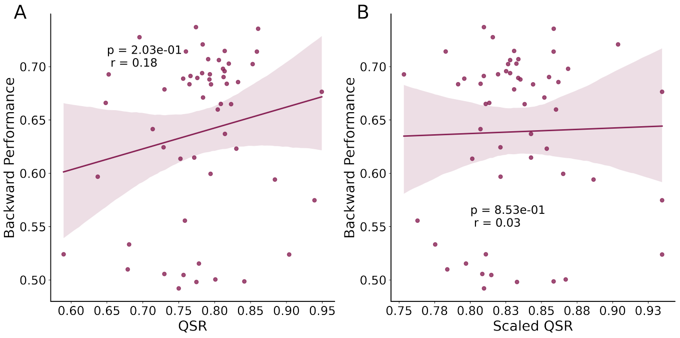

Fig O. Relationship with Quadratic Scoring rule, model-free measure of metacognitive sensitivity in high-variance condition of task. A) Backward performance was not significantly correlated with QSR. B) Backward performance was also not significantly correlated with scaled-QSR, which determines a linear scaling of empirical confidence values to maximize QSR. The dots in the plots above represent the corresponding estimations for each subject. Each dot in the above plots reflects the associated estimations for the subjects in the high variance condition of task.

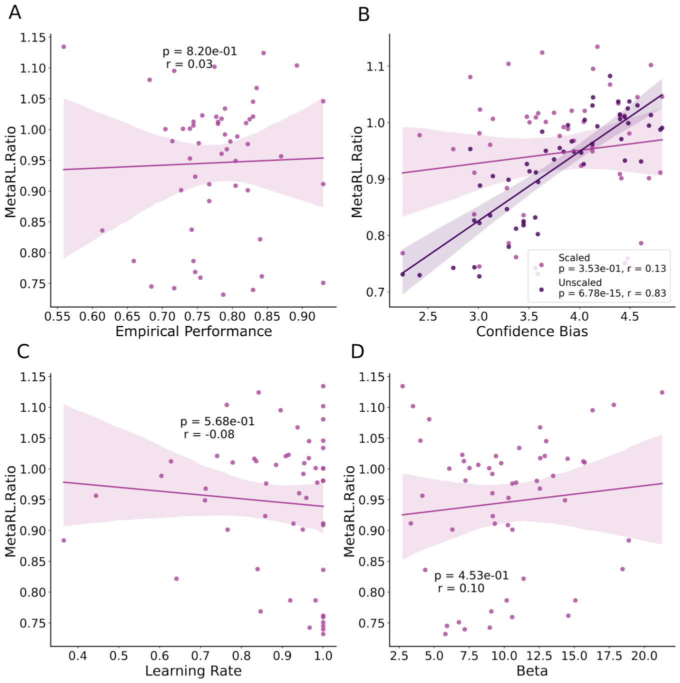

Fig P. Relationship between MetaRL.Ratio and empirical choice parameters in high-variance condition of task. A) The MetaRL.Ratio, our measure of metacognitive efficiency, was independent of empirical performance. B) The correlation between MetaRL.Ratio and confidence-bias decreased after applying the confidence scaling method. C & D) The MetaRL.Ratio was not significantly correlated with the inverse-temperature (C) or the learning-rate (D) of the Forward model. The above plots display dots that represent the relevant estimations for each subject in the high variance condition of task.

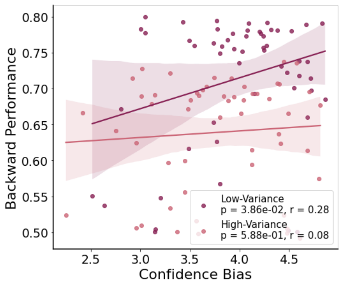

Fig Q. Relationship between Backward performance and confidence bias in low- and high-variance conditions. Backward performance was not independent of confidence bias in the low-variance condition, whereas it was independent in the high-variance condition.

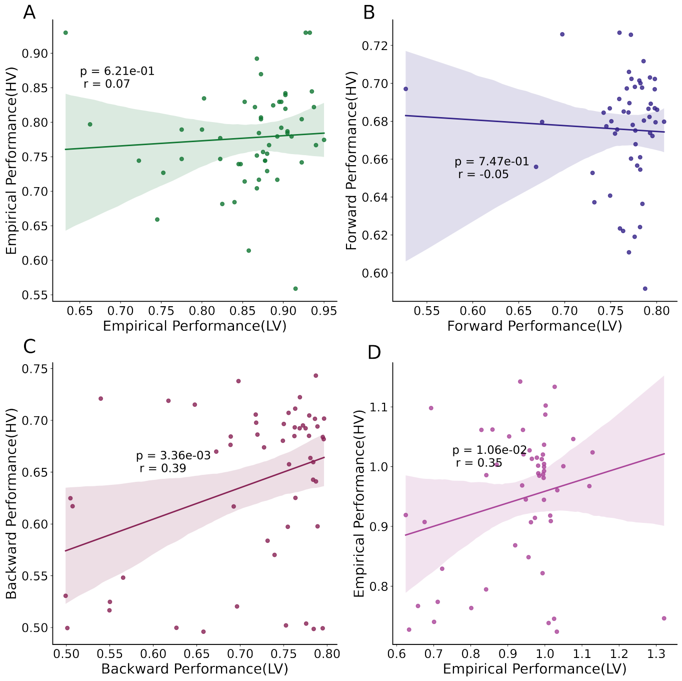

Fig R. Correlation of empirical performance and MetaRL.Ratio between low- and high-Variance Conditions. Empirical and Forward model performance were not correlated between the two task conditions. In contrast, both the Backward performance (red) and MetaRL.Ratio (purple) were.

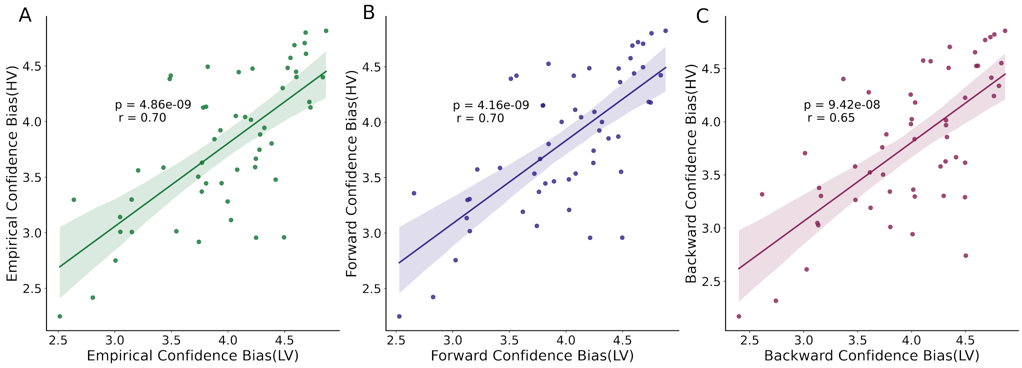

Fig S. Correlation of confidence-bias between Low- and High-Variance Conditions. The confidence-bias was correlated between two task difficulties for empirical data and also Forward and Backward models.

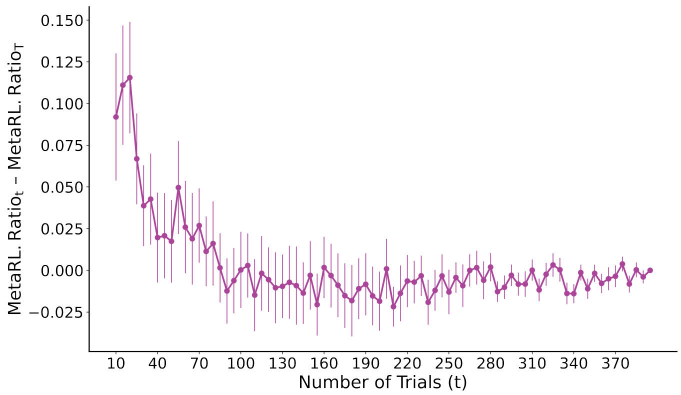

Fig T. The required number of trials for stability of our measure. The difference of MetaRL.Ratio across all trials, called MetaRL.Ratio$_T$, from MetaRL.Ratio$_t$ (the estimate of the MetaRL.Ratio from trial 10 to t (10 < t)) was utilized as an estimation of stability of our measure. The MetaRL.Ratio$_t$ got closer to MetaRL.Ratio$_t$ after trial 100 and the variance decreased considerably after trial 280, while there were 400 trials in the task.
