# Supplementary material for: Metacognitive efficiency in learned value-based choice
Source: PLoS Comput Biol. 2026 Mar 31;22(3):e1014108. doi: 10.1371/journal.pcbi.1014108 (PMC13102309; doi:10.1371/journal.pcbi.1014108)
Supplement: Attachment — Submitted filename: MetaRL,response.pdf [file pcbi.1014108.s004.pdf]

Reviewer #1: Review of "Metacognitive efficiency in learned value-based choice"
Reviewer: Michael Landy

This paper develops an analogue of M-ratio for a bandit choice task that the authors call MetaRL.Ratio. The analogy is needed because in this value-based task with changing conditions (the average payoffs swap between bandits every 20 trials or so) the participant's estimates of value are constantly changing so that, effectively, the d' for which machine is preferable is also constantly changing. The paper examines several competing models as well, and shows that MetaRL.Ratio has much of what would desire in a statistic that measures metacognitive efficiency independently of first-order sensitivity. I've worked some in metacognition in recent years, but not in RL, so I'll take the authors' word that work on metacognition for RL tasks is new. It's an excellent first effort in that direction: clear and thorough.

Thank you very much for this assessment and your comments.

My comments are mainly about clarity.

Specifics (page/para/line):

2/3/16: This is the first mention of QSR and of the Brier score. Since neither is defined here, mentioning that QSR is 1 minus the Brier score is kind of useless for the reader who is unfamiliar with either (which I was as I read it). Now that I know what they are, the description of the QSR as "model free" seems incorrect in the current context. The Brier score is a difference of estimated and actual probability (per trial here) and it seems to me that the "estimate" here can only come from a model of the participant's behavior, so not exactly model-free.

Apologies for not making this clear. We updated lines 73-75 to state:

QSR, which is a model-free measure of meta-cognitive sensitivity. This is computed as one minus the Brier score which, in turn, is the sum of the square difference between accuracy and reported confidence about choice across all trials.

The Brier score is the difference of reported confidence and accuracy. As I understood you called them estimated and actual probability respectively, am I right? If so, the reported confidence comes from empirical data and is not based on any parametric model of the decision-making, thus called model-free. It is also noticeable that reported confidence in our experiment was about confidence of subject about her choice. One may argue, that confidence about choice in a trial is not necessarily comparable with accuracy of choice in that trial. Thus, criticizing that QSR is based on an assumption that confidence and accuracy in each trial on comparable.

This is a good point. We completely agree that confidence on each trial could, in principle, be dependent on other parameters of decision-making, including exploration/exploitation, and this would disturb the relationship between accuracy and confidence. In fact, in a recent study (Solopchuk & Dayan, PLoS One, 2025), we showed that participants were extremely resistant to giving different answers to questions of the sort: 'How confident are you that the choice you made will give you the most immediate reward?' versus 'How confident are you that the choice you made will give you the most reward in the long run?'.

Our current model-based analysis of confidence judgements could include the effect of exploratory choices if the process in the 'backward' model correctly captured the mechanism that the participants used. For the moment, we have left this for future study, noting the Solopchuk result above. We put a comment about it in the Discussion:

**Discussion note**

4/1/4: The switches happen every 18-22 trials and you tell the participant that is the situation. Thus, an ideal-performance model would include counting trials and anticipating a change only starting with the 18th trial (and averaging the values up until then). After the first switch, the time of switch is uncertain, but similar ideal principles apply (somewhat analogous to my paper on categorization with randomly switching priors, with Weiji Ma, Elyse Norton and Luigi Acerbi). You mainly consider two variants of Q-learning as well as the QSR, all of which ignore this knowledge of the participants. I was a little surprised by the lack of discussion of possible models that take this knowledge into account.

We had sought to focus the paper on the issues of confidence rather than do a more extensive model comparison. However, to address your important point, we have now fitted a Hidden Semi-Markov Model (HSMM), which incorporates the belief about reversals, as well as a Mixture model that combines our model-free RL model with the HSMM model for decision-making. These updates can be found in the Methods section, lines 540–575.

**Model-Based decision-making; Hidden Semi Markov Model (HSMM).** In the Model-Based (MB) framework the latent state of the environment are inferred by using an HSMM that explicitly accounts for state durations. Here, the two latent states (indexed by $S \in \{1, 2\}$) represent, low-reward and high-reward options.

We denote the sequence of latent states as $S_{1:t}$ from time 1 to $t$. Let $h$ be the time already spent in the current state, and let $d$ be the total duration of the current state. Then we have:

$$\begin{aligned} &P(S_{t-h+1:t+d-h} = i,\, r_{1:t-1} \mid a_{1:t-1},\, \theta) = \\ &\quad P(r_{t-h+1:t-1} \mid S_{t-h+1:t-1} = i,\, a_{t-h+1:t-1},\, \theta) \\ &\quad P(S_{t-h]} = j,\, r_{1:t-h} \mid a_{t-h},\, \theta)\, P(d \mid \theta), \end{aligned} \tag{18}$$

where $\theta = (D, n, p, \mu_S^a, \sigma)$ is the tuple of parameters used to define the model, and $i \neq j$ since we only have two states. Moreover $a_{1:t-1}$ is the sequence of actions from trial 1 to $t-1$. Using marginalization, we obtain:

$$\begin{aligned} &P(S_t = i,\, r_{1:t-1} \mid a_{1:t-1},\, \theta) = \\ &\quad \sum_{d=1}^{D} P(d \mid \theta) \sum_{h=1}^{d} \alpha(t-h, j)\, P(r_{t-h+1:t-1} \mid S_{t-h+1:t-1} = i,\, a_{t-h+1:t-1},\, \theta), \end{aligned} \tag{19}$$

where $\alpha(t-h, j)$ is called the *forward variable* and is defined as the joint probability of the observed rewards from time 1 to $t-h$, denoted as $r_{1:t-h}$, and the event that state $j$ ended at time $t-h$. It can be computed recursively for any time $t$ and state $j$:

$$\alpha(t, j) = \sum_{d=1}^{D} \alpha(t-d, i)\, P(r_{t-d+1:t-1} \mid S_{t-d+1:t-1} = j,\, a_{t-d+1:t-1},\, \theta)\, P(d \mid \theta). \tag{20}$$

Duration Modeling. We assume that a latent state persists for a duration $d$ (with $d = 1, \ldots, D$, and in our implementation $D = 70$, as the maximum number of consecutive identical actions observed across subjects and conditions was 68). The duration is modeled by a negative binomial distribution:

$$P(d|\theta) = \binom{d+n-1}{d} p^n (1-p)^d, \tag{21}$$

where $n$ and $p$ are parameters.

Emission Model. This is the probability of a sequence of observed rewards given the latent states and actions in each trial $P(r_{t:t+d} \mid S_{t:t+d},\, a_{t:t+d},\, \theta))$. Since trials are independent:

$$P(r_{t:t+d} \mid S_{t:t+d},\, a_{t:t+d},\, \theta) = \prod_{k=t}^{t+d} P(r_k \mid S_k, a_k, \theta), \tag{22}$$

where $P(r_k \mid S_k, a_k, \theta)$ is modeled as a Gaussian distribution with the same mean ($\mu_S^a$) and variance ($\sigma^2$) as in the task. Without loss of generality, we assume that in state $S_t = 1$, action $a_t = 1$ yields a higher average reward, and the reverse holds for $S_t = 2$. Then we have:

$$P(r_t \mid s_t, a_t, \theta) = \begin{cases} \mathcal{N}(r_t; \mu_S^a = 60, \sigma^2) & \text{if } S_t = a_t, \\ \mathcal{N}(r_t; \mu_S^a = 40, \sigma^2) & \text{if } S_t \neq a_t, \end{cases} \tag{23}$$

where $\sigma^2$ depends on whether the task condition is high or low variance.

Using Eq. 19, we can compute the posterior probability of each state:

$$P(S_t \mid r_{1:t-1},\, a_{1:t-1}, \theta) = \frac{P(S_t, r_{1:t-1} \mid a_{1:t-1},\, \theta)}{\sum_{S'_t} P(S'_t, r_{1:t-1} \mid a_{1:t-1},\, \theta)} \tag{24}$$

Finally, we define the probability of each action corresponding to the probability of the state in which that action has a higher average reward. For example, if we set in state 0, the $a_t = 1$ has a higher reward, and in state 1, the $a_t = 2$ has a higher reward, then we have:

$$\begin{aligned} P_{MB}(a_t = 1 \mid S_t) &= P(S_t = 0 \mid r_{1:t-1},\, a_{1:t-1}, \theta) \\ P_{MB}(a_t = 2 \mid S_t) &= P(S_t = 1 \mid r_{1:t-1},\, a_{1:t-1}, \theta) \end{aligned} \tag{25}$$

For each subject, we performed a grid search over candidate negative binomial parameters $(n, p)$ by computing HSMM state probabilities over $T = 400$ trials and then calculating the negative log-likelihood (NLL) of the observed actions using a protected logarithm. The candidate with the lowest NLL was selected as the best-fit parameter.

**Mixture Model, combination of both MF and MB models.** The MB probabilities $P_{MB}(a|S)$ (obtained as shown above) are combined with the model-free (MF) probabilities computed via a softmax function over $Q$-values (Equation 8). In our simulation the overall choice probability is a weighted mixture:

$$P_{Mix}(a_t|S_t) = \gamma\, P_{MF}(a_t) + (1-\gamma)\, P_{MB}(a_t|S_t), \tag{26}$$

where $\gamma$ is the mixture weight.

Thus the MB probabilities are mixed with MF probabilities to simulate an action response, and the HSMM forward variable is updated with the new reward information for subsequent trials. The fitting method for the mixture model was the same as the one for the HSMM.

We also updated the “Results” section, lines, 325-361 in the updated draft, as below to present the  comparison between the new models and the basic MF RL models.

**Comparison with a Hybrid Model of Choice.** For simplicity, we introduced our framework was introduced based on model-free (MF) reinforcement learning (RL) methods. However, all these MF RL models performed significantly worse than participants, perhaps because the subjects were informed that reversals would occur every 18–22 trials. To capture human behavior more proficiently, we applied a Hidden Semi-Markov Model (which we refer to as being model-based or MB) and a Mixture Model, where choice probabilities combined MF and MB action values. As a forward model,  the MB fit to empirical choices was weaker than the MF (AIC = 295.44 vs. 224.23, W = 260, p = 3.261e-05; BIC = 303.42 vs. 232.22, W = 260, p = 3.261e-05). In contrast,  the  Mixture model fit was better than MF using AIC to penalize complexity (AIC = 193.26 vs. 224.23, W = 93, p = 2.24e-08). However, it did not fit better than MF based on BIC (BIC = 213.22 vs. 232.22, W = 93, p = 7.144e-01). Additionally, the Mixture model outperformed MB in explaining empirical choices (AIC = 193.26 vs. 295.44, W = 1, p = 1.72e-10; BIC = 213.22 vs. 303.42, W = 5, p = 2.153e-10) (see Supplementary Figure 23).  The performance of MB and Mixture models were significantly closer to empirical performance compared to MF (MB vs. MF: 0.87 vs. 0.76, W = 0, p = 1.626e-10; Mixture vs. MF: 0.85 vs. 0.76, W = 0, p = 1.626e-10). However, while the Mixture model’s performance was significantly different from empirical performance (M = 0.85 vs. 0.86, W = 312, p = 2.1e-04), MB performance was not significantly different from empirical performance ( M = 0.87 vs. 0.86, W = 686, p = 9.818e-01) (see Supplementary Figure 8). In summary, the Mixture model emerged as the best model, as its performance was closer to empirical data than that of MF, and its goodness-of-fit (as measured by both AIC and BIC) was superior to that of MB.

We also fitted the Mixture model to empirical choices and confidences as our Backward model. The Mixture model of confidence was fitted significantly better than the MF model of confidence (M = .68 vs .745 , W = 0, p = 3.504e-10). The MetaRL.Ratio based on the mixture model was highly correlated to QSR (model-free measure of meta-cognitive sensitivity) (r = .69, p = 8.45e-09) (see Supplementary Figure 26). Thus, the best choice model also resulted in a better confidence model and greater consistency with QSR. In addition, the MetaRL.Ratio was independent from accuracy (r = -.02, p = .886) while dependent on confidence-bias (r = .49, p = 1.56e-04).  Following the same approach as in our model-free analysis of confidence, we incorporated a leaky confidence term Lebreton et al. (2019) into the confidence representation of the Backward model. Although the leaky representation of confidence provided a better fit to empirical

choices and confidence ratings compared to the basic Backward model (M = .66 vs .68, W = 33.5, p = 1.56e-09), it still failed to make the MetaRL.Ratio independent of confidence bias in this case (r = .41, p = .002).

In the Supplementary Materials, we added Figures 7–11, which correspond to the results mentioned above, along with additional plots from the same analyses conducted for our MF model in the main paper. Specifically, Figures 7–8 present model comparison and performance comparison between the models and empirical data.

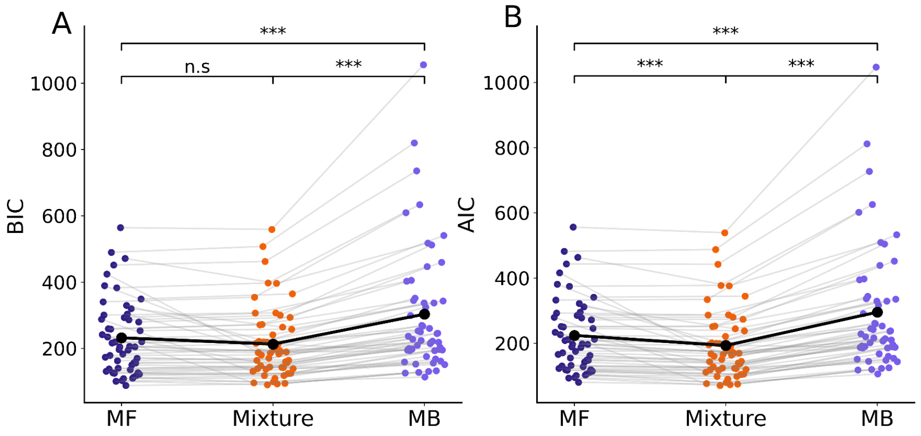


**Figure 7. Comparison between MF, Mixture model, and MB in the low-variance condition of task.** A) The goodness of fit, as measured by BIC, was not significantly different between MF and the Mixture model. However, both the Mixture model and MF fit better than the MB model. B) According to AIC, the Mixture model provided a better fit than both MF and MB, while MF fit our empirical data better than MB.

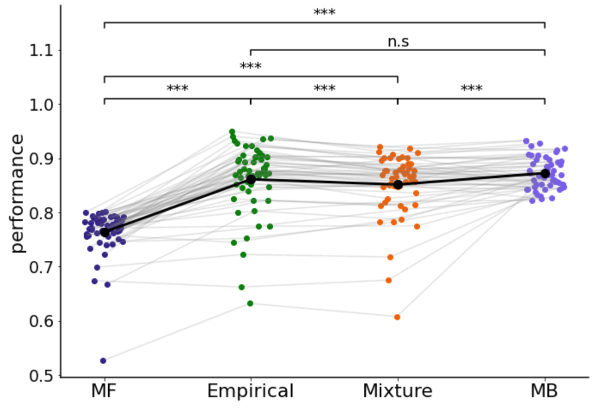

**Figure 8. Comparison between empirical performance and models performance in the low-variance condition of the task.**
The performance of both the Mixture model and MB was significantly different from MF and closer to empirical performance. However, the Mixture model's performance still deviated significantly from those of the empirical choices.

We also updated the relevant paragraph in the "Discussion" section, lines 363-366:

To enhance interpretability, we presented our main results based on a simple RL model of choices. However, we also demonstrated that the characteristics of our measures remain consistent when applying more advanced model-free RL and model-based (Hidden Semi-Markov) treatments of choice and confidence….

5/1/2: "worse choice Backward performance" doesn't parse. Maybe delete "choice"?

yes, thank you! we updated the line 157:

generate a worse Backward performance

5/4/4: I suppose you have to say "slightly higher" given a significant p-value (seems suspect, but so it goes...), but really a 1/4% difference is not significant (in the common English meaning of the word). Oy!

Thank you for pointing out the discrepancy between the statistical and practica significance of the results. We updated the lines 183-189 as:

However, the confidence bias of the Forward model was marginally (though statistically significantly) higher than the confidence bias of the subjects (M = 3.99 vs 3.98, W = 102, p = .017). The very small difference could come from our implementation of bias correction. To further quantify this, we compared the absolute differences between model-based and empirical confidence biases. The absolute difference between the Backward model and empirical ratings was significantly smaller than that between the Forward model and empirical ratings (M = 0.027 vs. 0.095, W = 82, p = 1.29e-08).

The supplementary figures are not in the order in which they are cited in the main text. Also Supp. 3F is cited before 3E.

Apologies. We reordered the plots in the supplementary materials and swapped the sentences referring to Supp. 3E and 3F.

6/4/5: you saw "The Scaled-QSR was not correlated...", but then cite a statistic that indicates that it was correlated. Delete "not"?

Sorry about that. We updated the line 226 to read:

The Scaled-QSR was correlated….

Figure 4 and other scatterplots: Are the dots individual subjects? Pairings of subjects and variance condition? Legends should clarify this throughout.

Yes, the dots across all the plots represent individual subjects. Following your comment, we added clarifying sentences to all the plots, including details about the task conditions. Figures 3, 4, and 5 pertain to subjects in the low-variance condition, while comparisons with the high-variance condition begin in Figure 6.

We updated the draft by adding the lines 159-160:

Unless otherwise stated, all results in this draft refer to low-variance conditions. Results for high-variance conditions are provided in the supplementary materials.

We also added the equivalent plots in high-variance condition to our supplementary materials, Figures 3, 4 and 5.

There were some differences between the low- and high-variance conditions in our findings:

A) Correlation Between QSR and Backward Performance: In the high-variance condition, the correlation between QSR and Backward performance was not significant ($r = 0.18$, $p = 0.19$), while in the low-variance condition, the correlation was significantly positive ($r = 0.5$, $p = 0.0001$). This suggests that MetaRL.Ratio was consistent with QSR in the low-variance condition but not in the high-variance condition.

B) Dependency of MetaRL.Ratio on Confidence-Bias: after scaling confidence, the MetaRL.Ratio was significantly dependent on confidence bias in the low-variance condition ($r = 0.35$, $p = 0.008$), while in the high-variance condition, this dependency was not significant ($r = 0.15$, $p = 0.2$).

To examine these results in more detail, we analyzed the relationship between backward performance and the confidence bias:

B*) Dependency of Backward performance on Confidence-Bias: the MetaRL.Ratio, after confidence scaling (Figure 5B), exhibited a significant correlation with confidence bias in the low-variance condition ($r = 0.28$, $p = 0.0386$). In contrast, no

significant relationship was found in the high-variance condition (r = 0.08, p = 0.588). These results are illustrated in Supplementary Figure 24.

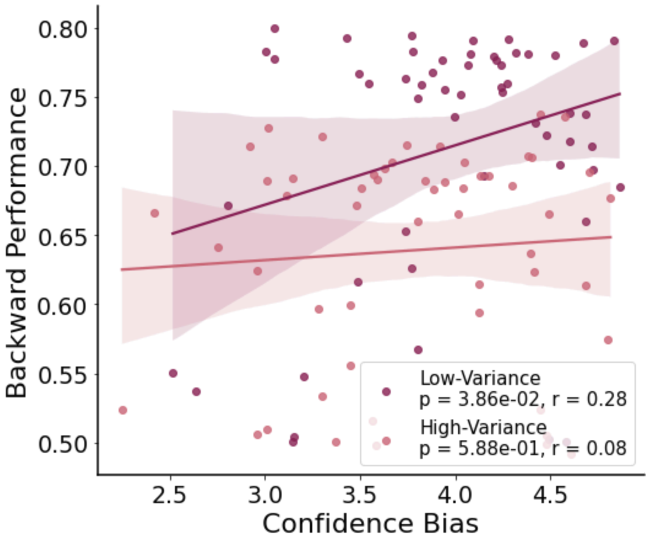


**Figure 24: Relationship between Backward performance and confidence bias in low- and high-variance conditions.** Backward performance was not independent of confidence bias in the low-variance condition, whereas it was independent in the high-variance condition.

We also reported in the main draft, lines 222–224: “A prominent issue with QSR is that it depends on confidence bias. This is evidenced by a significant correlation between the two (r = 0.77, p = 1.13e-11).” In addition, we observed that confidence bias was significantly higher in the low-variance condition compared to the high-variance condition (M = 3.98 vs. 3.79, W = 400, p = .003), as shown in Figure 6 of the main paper.
Therefore, further analyses are needed to clarify whether confidence bias contributes to the observed correlation between QSR and Backward performance in the low-variance condition. Exploring differences between the low- and high-variance conditions may be a fruitful direction for future research.

8/1/4: I would vote for dropping the scientific notation for p-values when they are larger than .001 (as in "p=.06").

Good idea! We have updated them in line with your suggestion.

8/2/8: hovered around a value of 1 AS WE VARIED Learning-rate...

Line 253:
The MetaRL.Ratio consistently hovered around a value of 1 as we varied the Learning-rate

9/2/1: was higher in THE high- versus THE low-variance condition

Line 280:
was higher in the high- versus the low-variance condition

9/last sentence: Much of this page feels like a data dump rather than a description of results that impact a previously stated hypothesis. This last sentence is, to me, a non-conclusion conclusion (it doesn't say what the results mean concerning value-based meta-cognition, but only that you were able to measure stuff).

Thank you for your feedback. We removed the sentence, as the related content is already covered in the Discussion section.

Figure 6: The legend describes green vs. blue backwards from the axis labels in the plot, and the key is really obscure for figuring out which violin plots correspond to low vs. high variance. I suggest you put the words "Low" and "High" below the corresponding columns and maybe "Variance:" to the left.

Thank you for the suggestion. We updated Figure 6 by placing "LV" and "HV" under each corresponding violin plot. Since there wasn't enough space to write the full labels, we introduced the abbreviations in the legend.

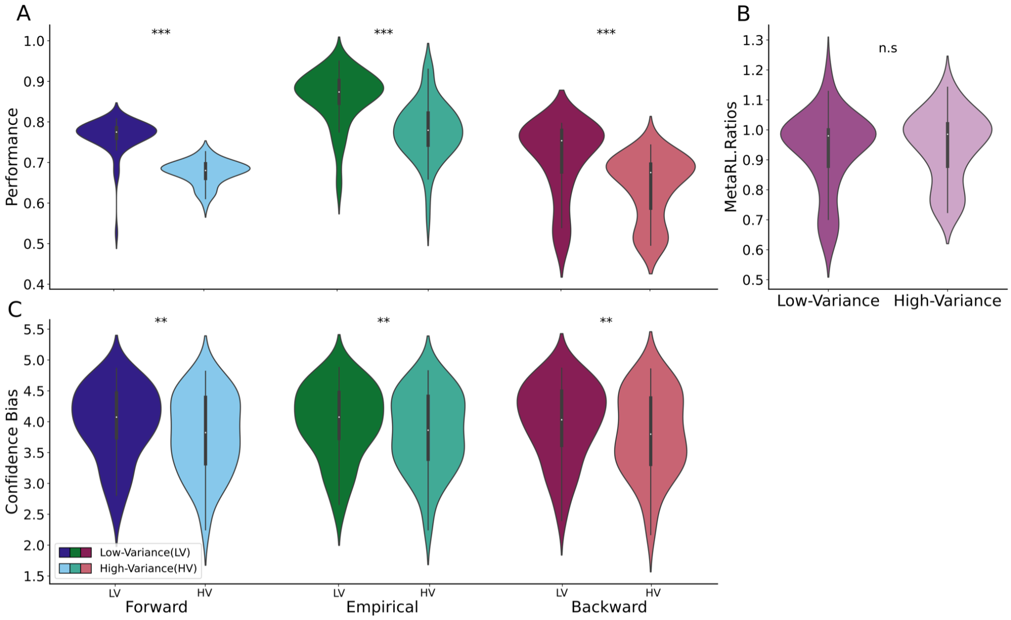


Figure 6: **Independence of MetaRL.Ratio from task difficulty.** A) Empirical performance (green), Forward performance (blue) and Backward performance (red) were significantly lower in the high- than the low-variance condition. B) The MetaRL.Ratio was not significantly different between the two levels of task difficulty. C) The confidence-bias was significantly lower in high-variance rather than low-variance condition of task according to empirical data (green), Forward model (blue) and Backward model (red).

Tables 1 and 2: The values in the tables all (mostly?) appear in the running text,

making the tables (almost) redundant. I don't understand why these correlations didn't deserve a scatterplot (possibly in the Supplement if you prefer) but all the other correlations did.

Thank you for your point, we added the tables to summarize the information that was in the text so that the results can more easily be grasped, but we have now also added scatter plots to the supplementary Figures 19 and 20.

12/2: Is the Supplement figure corresponding to this paragraph ever cited in the main text? I didn't check to make sure that all Supplementary figures were cited.

Thank you for your question. We have now ensured that all the Supplementary figures are cited – some of them in the Discussion section.

14: There were a lot of experimental details omitted from the Methods section: description of the participants (how many, age, etc.), informed consent, what they were paid and whether payment was increased in some way by single-trial or overall RL performance and details of the trial-by-trial feedback.

Sorry – we had left them out since ours is not the primary report of this experiment. However, you are quite right that the information should be sufficient. We have updated the "Materials and Methods" section accordingly, lines 485-509:

**Cognitive task.** The experiment included sixty participants, all of whom were first-year psychology students at KU Leuven participating for course credits. Data from six participants were removed because their performance remained at chance level in at least one of the blocks, leaving a final sample of fifty-four participants. Participants completed the task on their own PC, with data collected online via the Pavlovia platform (pavlovia.org). Only those using a Windows PC with an external mouse were allowed to take part. They were informed that the goal of the task was to earn as much money as possible on virtual slot machines. On each trial, participants chose between two slot machines-mapped to the S-key (left) and F-key (right)-with one machine offering a higher average reward. They were told that every 18-22 trials, a switch would occur so that the previously worse slot machine would become the better one. After each choice, participants indicated their confidence on a continuous scale ranging from "this was a guess" to "very certain." A numerical reward based on their choice was then displayed, and the next trial commenced.

In the low-variance condition of the experiment, the rewards following a worse or better choice were drawn from normal distributions N(40, 8) or N(60, 8). In the high-variance condition, rewards were drawn from
N(40, 16) or N(60, 16). These conditions were administered in a counterbalanced order, 12 to 72 hours apart, and each was followed by a medium-variance condition that is not analyzed here. Both the low- and high-variance conditions comprised 20 blocks of 18-22 trials, for a total of 400 trials in each condition.

The three highest-scoring participants received a gift voucher from a local store. Information about age, gender, and handedness was not recorded and is therefore unknown, although one can typically expect a majority of right-handed, 18-year-old, mostly female participants in this pool. All participants provided informed consent via e-mail and were unaware of the hypotheses of the study. The study was approved by the Social and Societal Ethics Committee (SMEC) of KU Leuven.

14/5/5: that we do NOT analyze

Thank you, We changed it.

15/1: I can't guess what this chi-squared test is nor what criterion you use on its results to merit exclusion.

Sorry it was unclear, we updated lines 510-515 as follows:

**Exclusion criteria.** To exclude participants performing at or below chance level, we applied a Chi-Square Goodness-of-Fit test separately to the high- and low- variance conditions. This method compares the observed performance (correct vs. incorrect responses) to the expected distribution under random guessing (50% correct). The Chi-Square statistic was calculated as:

$$\chi^2 = \sum \frac{(O_i - E_i)^2}{E_i} \qquad (1)$$

where $O_i$ is the observed frequency and $E_i$ is the expected frequency under the null hypothesis. We used 1 degree of freedom because the test involves two categories (correct and incorrect responses), with the degrees of freedom calculated as the number of categories minus one. Participants whose performance produced a chi-square value exceeding the critical threshold of 3.841 at 1 degree of freedom ($p < 0.05$) were excluded from further analysis. This resulted in the exclusion of six participants.

15/3/after Eq. 3: "when run autonomously on the task using the same (potential) reward sequences as the participants": I found this unclear. The RL algorithm is deterministic, so I'm guessing that you re-run the model on sequences of pairs of payoffs that are newly sampled from the appropriate Gaussian distributions and do that a bunch of times to get average performance, etc. BUT, the quoted sentence is unclear to me whether it means you ran the model ("?autonomously") only on the sequence the participant experienced or on new ones.

The RL algorithm is stochastic, as it uses the soft-max function with temperature parameter to make choices. We ran the RL algorithm on the sequence the participants experienced. We changed the sentence to clarify this, lines 520-525 in the main draft:

We evaluated the accuracy of the best-fitting Forward model according to the frequency with which it chose the option with higher average reward, PerfF*s* for participant *s*, when run autonomously on the task using the same (potential) reward sequences as the participants experienced in the experiment. This is as we would implement for a posterior predictive check of the fit of a model.

Eqs. 8-9: First, I found the jump from H/L in Eq. 8 to H^{B,s}/L^{B,s} a bit jarring. H/L sounds like a fixed pair of numbers (the highest and lowest confidence the subjects used), whereas H^{B,s}/L^{B,s} sound like free parameters (possibly constrained to be at least as wide as the participant's range?).

Sorry. In Eq. 9, H/L are actually parameters in the range [0,1] subject to the condition that L < H., In Eq. 10, we used an optimization algorithm to determine the values of H^{B,s}/L^{B,s} (which were fixed numbers) that minimized the mean quadratic distance between the model's, and each subject's, confidence reports. For clarity, we updated the text, the new text is available in response to the below question.

Also, you call the cost function "maximum likelihood", which implies you are thinking of additional Gaussian noise on produced confidence settings. Gaussian noise is a bit sketchy, since the range is constrained.

Sorry for the unclear description of our method of fitting. Indeed, we were just minimizing the Euclidean distance. We edited the text as follows:

In full:

$$Q_t(a_t) = Q_{t-1}(a_t) + \alpha(r_t - Q_{t-1}(a_t)) \quad (7)$$

$$P_t(a_t = i; \alpha, \beta) = \frac{\exp(\beta * Q_t(i))}{\exp(\beta * Q_t(1)) + \exp(\beta * Q_t(2))} \quad \text{for } i \in \{1, 2\} \quad (8)$$

and, writing this as $P_t^{B,s}(a_t^s)$ for the backwards model for participant $s$, the modeled confidence is:

$$\text{Conf}_t^{B,s}(L, H) = (H - L) * P_t^{B,s}(a_t^s) + L \quad (9)$$

in which $L$ and $H$, $L < H$, were parameters with the same restriction as the equivalent parameters in the Forward model. The parameters were fitted by minimzing the Euclidean distance between the confidence values predicted by the model and those reported by the subjects:

$$(\alpha^{B,s}, \beta^{B,s}, L^{B,s}, H^{B,s}) \sim \arg \min_{\alpha,\beta,L,H} \sum_{t=1}^{N} \left(\text{Conf}_t^{B,s}(L, H) - \text{Conf}_t^s\right)^2 \quad (10)$$

noting the dependence of $P_t^{B,s}(a_t^s)$, and thus $\text{Conf}_t^{B,s}$ on $\alpha, \beta$.

We also updated the text explaining the Forward model:

To model Forward confidence, we scaled the output of the softmax (written as $P_t^{F,s}(a_t^s)$ for participant $s$) using parameters $L$ and $H$, both in the range $[0, 1]$ and subject to $L < H$:

$$\text{Conf}_t^{F,s}(L, H) = (H - L) * P_t^{F,s}(a_t^s) + L \quad (5)$$

which we fit by minimizing the Euclidean distance between the scaled and empirical ($\text{Conf}_t^s$) confidence (using scipy.optimize.minimize).

$$(L^{F,s}, H^{F,s}) \sim \arg \min_{L,H} \sum_{t=1}^{N} \left(\text{Conf}_t^{F,s}(L, H) - \text{Conf}_t^s\right)^2 \quad (6)$$

16/3 (Fittings and simulations): The explanation for 120 fits and 100 simulations is unclear. Are 120 fits done to try to find a global minimum (and not get stuck in local minima)? Is this non-convex?

Thank you for your comment. Yes, the 120 fits were performed to reduce the risk of getting trapped in local minima and to increase the likelihood of finding a good approximation of the global minimum. The optimization landscape is indeed **non-convex**, and we observed variability in fitted parameters depending on initialization. Empirically, we found that after 120 fits (with different random seeds

or initializations), the fitted parameters converged to stable values across participants. Therefore, we used this number of fits throughout our analyses.

Are the 100 simulations because you are, in fact, re-drawing the random payoffs on every simulation? Please clarify.

We ran the simulations 100 times because of randomness in choices of model, based on its softmax. As noted above in the response, the payoffs were not random, they were the same pay-offs that the subjects either experienced, or would have, depending on their choices.

We have revised the draft to improve clarity, line 530-534:

The fitting for all the analyses described in the ”Results” section or in ”Supplementary Materials” was performed 120 times to stabilize the estimates. Using the fitted parameters for each subject, we then averaged over 100 simulations to estimate model performance. This averaging accounts for the randomness inherent in the models due to the softmax choice function.

Eq. 10: For me, the \chi_{a=b} notation is non-standard. I might have used Kronecker \delta instead, but would have explained what it meant. Please clarify.

Sorry for the confusion. We have replaced this by an explicit condition:

**Scaled-QSR.** Given empirical choices $a_t^s$ (when the better bandit is $b_t^s$), the trial by trial accuracy was 1 if the empirical choice was the better bandit and 0 otherwise.

$$I_t^s = \begin{cases} 1, & \text{if } a_t^s = b_t^s, \\ 0, & \text{if } a_t^s \neq b_t^s. \end{cases} \tag{11}$$

The confidence values were $\text{Conf}_t^s$ for participant $s$ and the normal QSR was

$$\text{QSR}^s = 1 - \frac{1}{N} \sum_{t=1}^{N} \left( \frac{\text{Conf}_t^s - 1}{4} - I_t^s \right)^2 \tag{12}$$

Eqs. 11-12: Something seems wrong here. Conf_t^s is the empirical response (ranging from 1 to 4), so the normalization in Eq. 10 scales it to look range from 0 to 1. It makes no sense to then scale it in Eq. 11 and in Eq. 4. It sounds like you want, in Eq. 11, Conf_t^s to be a number that ranges from zero to one, i.e., you want to redefine it from what it was in Eq. 10. Please fix.

Sorry for the confusion. We should first clarify that in our updated draft the previous Eqs. 11-12 are now Eqs. 12-13. Regarding your comment, accuracy is already on a [0, 1] scale, whereas, in this study, Confidence was reported on a scale of [1,5]. Normalizing confidence ratings to the same range ensures that both metrics are comparable in magnitude. Without normalization, if confidence ratings had a different scale (e.g., [1, 6] or [0, 100]), the quadratic scoring rule (QSR) calculation could overweight or underweight one metric relative to the other.

**Scaled-QSR.** Given empirical choices $a_t^s$ (when the better bandit is $b_t^s$), the trial by trial accuracy was 1 if the empirical choice was the better bandit and 0 otherwise.

$$I_t^s = \begin{cases} 1, & \text{if } a_t^s = b_t^s, \\ 0, & \text{if } a_t^s \neq b_t^s. \end{cases} \tag{11}$$

The confidence values were $\text{Conf}_t^s$ for participant $s$ and the normal QSR was

$$\text{QSR}^s = 1 - \frac{1}{N}\sum_{t=1}^{N}\left(\frac{\text{Conf}_t^s - 1}{4} - I_t^s\right)^2 \tag{12}$$

In the above equation, the confidence which was in the range [1,5] was normalized to be comparable with accuracy. Without normalization, if confidence ratings had a different scale (e.g., [1, 6] or [0, 100]), the quadratic scoring rule (QSR) calculation could overweight or underweight one metric relative to the other. Then, we scaled the empirical confidence values according to the same procedure as for the Forward and Backward models (Equations 5-9):

$$\text{Conf}_t^{Q,s}(L, H) = (H - L) * \text{Conf}_t^s + L \tag{13}$$

and determined confidence bounds by finding

$$(L^{Q,s}, H^{Q,s}) \sim \arg\max_{L,H} 1 - \frac{1}{N}\sum_{t=1}^{N}\left(\frac{\text{Conf}_t^{Q,s}(L,H) - 1}{4} - I_t^s\right)^2 \tag{14}$$

We call the maximum value of the expression on the right of Equation 14 the scaled-QSR.

Supp Fig. 3 legend/12: "aligning with our expectations". Maybe I'm confused, but this seems silly; the Forward performance has to remain constant as a function of noise SD, so treating that as meeting an expectation is a tautology, no?

Yes - sorry – we phrased this awkwardly. The performance of the Forward model is indeed independent of this noise. We updated the text accordingly to clarify our intended point:

"We repeated the same procedure for simulated choices and confidence ratings coming from the Forward model fit to the same empirical data. Similar to our findings with the Hmeta(σ2) agent, we observed a decrease in Backward

performance under the influence of confidence noise, as expected, while Forward performance remained constant, as it had to."

Supp 4/2/1: Please define the term "Forward(\sigma^2) agent" before you use it (and likewise for the other agents).

We updated the text, the lines are at Supp 4/1/1:

We also explored the effect of confidence noise variance by fitting the Backwards model to the corrupted first-order confidence reports of a Forward($\sigma^2$) agent (with the $\sigma^2$ indicating the variance of the added noise).

Supp Fig. 10 and elsewhere: Clarify that a pair of dots is a subject, or whatever, in the legend. The long sentence in the legend doesn't parse.

Yes, you are correct. We updated all the captions by adding sentences such as:

"The dots in the plots above indicate the respective estimated values for each subject."

Figure 14: It seems silly to talk about "confidence noise with a standard deviation of 0" rather than to simply call is a noiseless model.

Yes, thank you for your consideration. We updated the text accordingly, the updated Figure address is Supp Fig. 18:

"Specifically, in the H-meta agent, these two Backward parameters exhibited a negative correlation for the noiseless model."

Reviewer #2: The manuscript presents a novel method for analyzing metacognitive efficiency in learning tasks. Traditional analysis methods are challenging to apply to these tasks, where choice difficulty changes dynamically. To address this, the authors have developed a measure called MetaRL.Ratio, analogous to the M-ratio measure    as it is defined by contrasting confidence-data-driven model performance with choice-data-driven model performance. This work has significant potential, broadening the scope of metacognitive studies to the learning domain, including future applications in the evaluation of non-human agent behavior. Below, I have several comments that would be considered for further improvement of the manuscript.

Thank you very much for your review and advice.

1. The proposed models exhibit worryingly high misfit to choice accuracy. This is concerning, as the validity of the proposed metacognitive measure is limited if the models do not successfully mimic the true generative process. The supplementary material reports the behavior of more complex models, but their choice accuracy does not appear to be presented. In any case, a more careful treatment of the misfit is expected.

Thank you for your valuable note. We added a description of the Hidden Semi-Markov Model (HSMM), which incorporates information about reversals, as well as a Mixture model that combines our model-free RL account with the HSMM. The later model performed best in terms of matching empirical performance and fitting scores. These updates can be found in the Methods section, lines 539–590.

**Model-Based decision-making; Hidden Semi Markov Model (HSMM).** In the Model-Based (MB) framework the latent state of the environment are inferred by using an HSMM that explicitly accounts for state durations. Here, the two latent states (indexed by $S \in \{1, 2\}$) represent, low-reward and high-reward options.

We denote the sequence of latent states as $S_{1:t}$ from time 1 to $t$. Let $h$ be the time already spent in the current state, and let $d$ be the total duration of the current state. Then we have:

$$\begin{aligned} &P(S_{t-h+1:t+d-h} = i,\, r_{1:t-1} \mid a_{1:t-1},\, \theta) = \\ &\quad P(r_{t-h+1:t-1} \mid S_{t-h+1:t-1} = i,\, a_{t-h+1:t-1},\, \theta) \\ &\quad P(S_{t-h]} = j,\, r_{1:t-h} \mid a_{t-h},\, \theta)\, P(d \mid \theta), \end{aligned} \tag{18}$$

where $\theta = (D, n, p, \mu_S^a, \sigma)$ is the tuple of parameters used to define the model, and $i \neq j$ since we only have two states. Moreover $a_{1:t-1}$ is the sequence of actions from trial 1 to $t-1$. Using marginalization, we obtain:

$$\begin{aligned} &P(S_t = i,\, r_{1:t-1} \mid a_{1:t-1},\, \theta) = \\ &\sum_{d=1}^{D} P(d \mid \theta) \sum_{h=1}^{d} \alpha(t-h, j)\, P(r_{t-h+1:t-1} \mid S_{t-h+1:t-1} = i,\, a_{t-h+1:t-1},\, \theta), \end{aligned} \tag{19}$$

where $\alpha(t-h, j)$ is called the *forward variable* and is defined as the joint probability of the observed rewards from time 1 to $t-h$, denoted as $r_{1:t-h}$, and the event that state $j$ ended at time $t-h$. It can be computed recursively for any time $t$ and state $j$:

$$\alpha(t, j) = \sum_{d=1}^{D} \alpha(t-d, i)\, P(r_{t-d+1:t-1} \mid S_{t-d+1:t-1} = j,\, a_{t-d+1:t-1},\, \theta)\, P(d \mid \theta). \tag{20}$$

Duration Modeling. We assume that a latent state persists for a duration $d$ (with $d = 1, \ldots, D$, and in our implementation $D = 70$, as the maximum number of consecutive identical actions observed across subjects and conditions was 68). The duration is modeled by a negative binomial distribution:

$$P(d|\theta) = \binom{d+n-1}{d} p^n (1-p)^d, \tag{21}$$

where $n$ and $p$ are parameters.

Emission Model. This is the probability of a sequence of observed rewards given the latent states and actions in each trial $P(r_{t:t+d} \mid S_{t:t+d},\, a_{t:t+d},\, \theta))$. Since trials are independent:

$$P(r_{t:t+d} \mid S_{t:t+d},\, a_{t:t+d},\, \theta) = \prod_{k=t}^{t+d} P(r_k \mid S_k, a_k, \theta), \tag{22}$$

where $P(r_k \mid S_k, a_k, \theta)$ is modeled as a Gaussian distribution with the same mean ($\mu_S^a$) and variance ($\sigma^2$) as in the task. Without loss of generality, we assume that in state $S_t = 1$, action $a_t = 1$ yields a higher average reward, and the reverse holds for $S_t = 2$. Then we have:

$$P(r_t \mid s_t, a_t, \theta) = \begin{cases} \mathcal{N}(r_t; \mu_S^a = 60, \sigma^2) & \text{if } S_t = a_t, \\ \mathcal{N}(r_t; \mu_S^a = 40, \sigma^2) & \text{if } S_t \neq a_t, \end{cases} \tag{23}$$

where $\sigma^2$ depends on whether the task condition is high or low variance.

Using Eq. 19, we can compute the posterior probability of each state:

$$P(S_t \mid r_{1:t-1},\, a_{1:t-1}, \theta) = \frac{P(S_t, r_{1:t-1} \mid a_{1:t-1},\, \theta)}{\sum_{S_t'} P(S_t', r_{1:t-1} \mid a_{1:t-1},\, \theta)} \tag{24}$$

Finally, we define the probability of each action corresponding to the probability of the state in which that action has a higher average reward. For example, if we set in state 0, the $a_t = 1$ has a higher reward, and in state 1, the $a_t = 2$ has a higher reward, then we have:

$$\begin{aligned} P_{MB}(a_t = 1 \mid S_t) &= P(S_t = 0 \mid r_{1:t-1},\, a_{1:t-1}, \theta) \\ P_{MB}(a_t = 2 \mid S_t) &= P(S_t = 1 \mid r_{1:t-1},\, a_{1:t-1}, \theta) \end{aligned} \tag{25}$$

For each subject, we performed a grid search over candidate negative binomial parameters $(n, p)$ by computing HSMM state probabilities over $T = 400$ trials and then calculating the negative log-likelihood (NLL) of the observed actions using a protected logarithm. The candidate with the lowest NLL was selected as the best-fit parameter.

**Mixture Model, combination of both MF and MB models.** The MB probabilities $P_{MB}(a|S)$ (obtained as shown above) are combined with the model-free (MF) probabilities computed via a softmax function over $Q$-values (Equation 8). In our simulation the overall choice probability is a weighted mixture:

$$P_{Mix}(a_t|S_t) = \gamma\, P_{MF}(a_t) + (1-\gamma)\, P_{MB}(a_t|S_t), \tag{26}$$

where $\gamma$ is the mixture weight.

Thus the MB probabilities are mixed with MF probabilities to simulate an action response, and the HSMM forward variable is updated with the new reward information for subsequent trials. The fitting method for the mixture model was the same as the one for the HSMM.

We also updated the “Results” section with below lines , 325-361 in the updated draft, for comparison between the above models and the basic MF RL models.

**Comparison with a Hybrid Model of Choice.** For simplicity, we introduced our framework was introduced based on model-free (MF) reinforcement learning (RL) methods. However, all these MF RL models performed significantly worse than participants, perhaps because the subjects were informed that reversals would occur every 18–22 trials. To capture human behavior more proficiently, we applied a Hidden Semi-Markov Model (which we refer to as being model-based or MB) and a Mixture Model, where choice probabilities combined MF and MB action values. As a forward model, the MB fit to empirical choices was better than the MF (AIC = 295.44 vs. 224.23, W = 260, p = 3.261e-05; BIC = 303.42 vs. 232.22, W = 260, p = 3.261e-05). The Mixture model also fit better than MF using AIC to penalize complexity (AIC = 193.26 vs. 224.23, W = 93, p = 2.24e-08). However, it did not fit better than MF based on BIC (BIC = 213.22 vs. 232.22, W = 93, p = 7.144e-01). Additionally, the Mixture model outperformed MB in explaining empirical choices (AIC = 193.26 vs. 295.44, W = 1, p = 1.72e-10; BIC = 213.22 vs. 303.42, W = 5, p = 2.153e-10) (see Supplementary Figure 23). The performance of MB and Mixture models were significantly closer to empirical performance compared to MF (MB vs. MF: 0.87 vs. 0.76, W = 0, p = 1.626e-10; Mixture vs. MF: 0.85 vs. 0.76, W = 0, p = 1.626e-10). However, while the Mixture model’s performance was significantly different from empirical performance (M = 0.85 vs. 0.86, W = 312, p = 2.1e-04), MB performance was not significantly different from empirical performance ( M = 0.87 vs. 0.86, W = 686, p = 9.818e-01) (see Supplementary Figure 24). In summary, the Mixture model emerged as the best model, as its performance was closer to empirical data than that of MF, and its goodness-of-fit (as measured by both AIC and BIC) was superior to that of MB.

We also fitted the Mixture model to empirical choices and confidences as our Backward model. The Mixture model of confidence fitted significantly better than the MF treatment of confidence ( M = 0.68 vs. 0.745 , W = 0, p = 3.504e-10). The MetaRL.Ratio based on the mixture model was highly correlated with our model-agnostic measure of meta-cognitive sensitivity (QSR) (r = 0.69, p = 8.45e-09; see Supplementary Figure 26). Thus, the better choice model also resulted in a better confidence model and greater consistency with QSR. In addition, the MetaRL.Ratio based on the Mixture model was independent of accuracy (r = -0.02, p = 8.86e-01), although still dependent on confidence-bias (r = 0.49, p = 1.56e-04) (See supplementary Figure 11 A;B). Following the same approach as in our model-free analysis of confidence, we incorporated a leaky confidence term Lebreton et al. (2019) into the confidence representation of the Backward model. Although the leaky representation of confidence provided a better fit to empirical

choices and confidence ratings compared to the basic Backward model (M = .66 vs. .68, W = 33.5, p = 1.56 × $10^{-9}$), it still failed to make the MetaRL.Ratio independent of confidence bias in this case (r = .41, p = 2.12 × $10^{-3}$).

In the Supplementary Materials, we have added Figures 7–11, which correspond to the results mentioned above, along with additional plots from the same analyses conducted for our MF model in the main paper. Specifically, Figures 7–8 present model comparison and performance comparison between the models and empirical data.

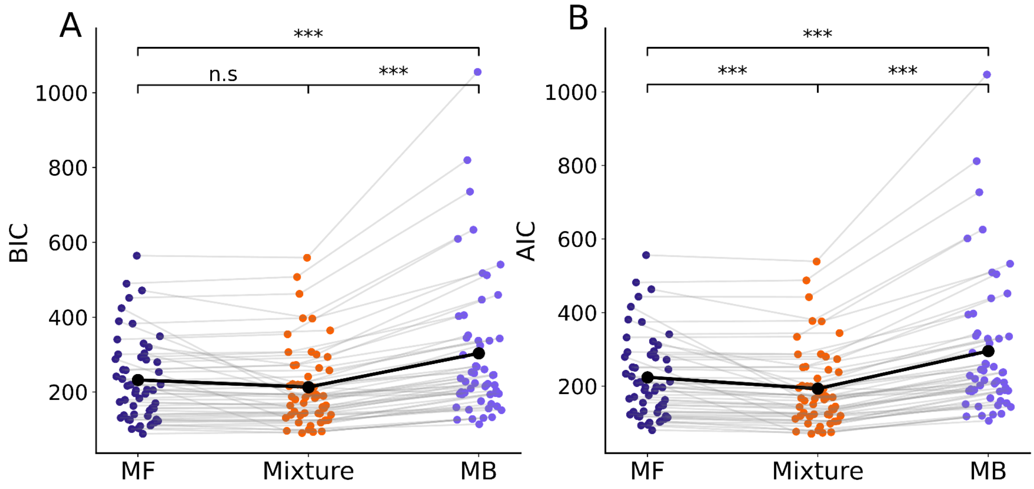


**Figure 7. Comparison between MF, Mixture model, and MB in the low-variance condition of task.** A) The goodness of fit, as measured by BIC, was not significantly different between MF and the Mixture model. However, both the Mixture model and MF fit better than the MB model. B) According to AIC, the Mixture model provided a better fit than both MF and MB, while MF fit our empirical data better than MB.

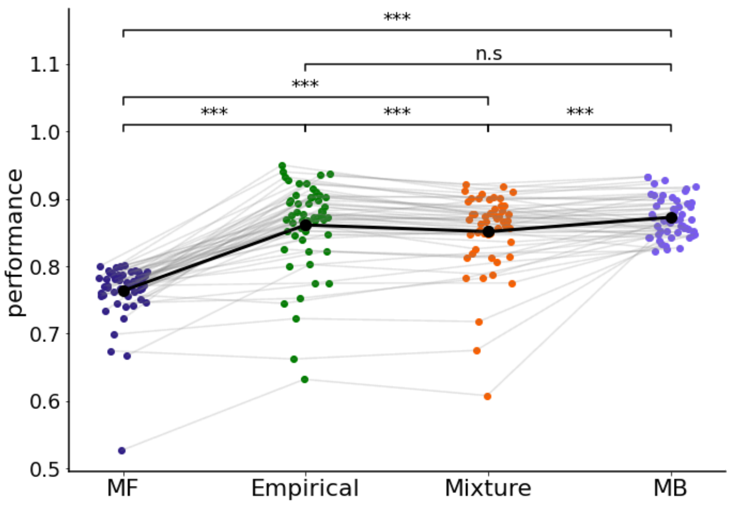

**Figure 8. Comparison between empirical performance and models performance in the low-variance condition of the task.**
The performance of both the Mixture and MB models was significantly different from the MF model and closer to empirical performance. However, the Mixture model's performance still showed a significant deviation from empirical choices.

We also updated the below paragraph in the “Discussion” section, lines 381-383:

To enhance interpretability, we presented our main results based on a simple RL model of choices. However, we also demonstrated that the characteristics of our measures remain consistent when applying more advanced model-free RL and model-based (Hidden Semi-Markov) models of choice and confidence….

2. On page 5, regarding the misfit of the backward model to choice accuracy, the authors state, “This was expected since the Backward model is fitted to make good predictions of confidence ratings, not choices.” I wonder if this is correct, since choice seems implicitly considered in equations 8 and 9 during the fitting of confidence data. Further methodological clarification around this point would be appreciated.

It is correct that choice is implicitly considered. However, although the Forward model is optimized to fit the choices, the Backwards model is not, but rather only the confidence reports.

Consider the unrealistic case that a subject chose the same bandit on every trial, but correctly reported high confidence in the choice when that bandit was actually better; and low confidence in the choice when it was worse. Then the Forward model would perfectly fit the choice, whereas the Backward model would be perfectly able to optimize the return from the bandit.
Conversely, for a subject whose confidence reports were first order probabilities from the Forward model that underlay their choices, we would expect Forward and Backward models to predict choices equally well.

3. The present report includes the misfit of accuracy and the presumably undesirable correlation between MetaRL.Ratio and confidence bias. It seems the authors used the present simplified model as a provisional measurement model for performance evaluation. However, the proposed framework could also be applied to a more sophisticated process model, which is expected to better represent human data generation mechanisms. I would like the authors to offer a perspective or guideline on the applications they recommend.

We appreciate this comment. We originally concentrated on very simple models of choice and confidence in the manuscript in order to focus on the main point about Forward and Backward models. In response to your first question, we constructed the Mixture model, which accommodates information about the possibility of reversals, and indeed fit the behavior more proficiently. Indeed, it is possible to use the framework of forwards and backwards fitting with much more complicated and sophisticated models - employing standard methods of model comparison to choose.

Below, we describe a potential method to decrease the dependency on confidence bias.

Given the dependence of MetaRL.Ratio on confidence bias under low variance condition, we proposed an improved 'Leaky' model of confidence which, as in previous studies (Lebreton et al., 2019; Salem Garcia et al., 2022), captures autocorrelation in confidence reports by using a weighted average of the ‘true’ confidence on the current trial and the confidence report on the previous trial. When using this leaky model, the MetaRL.Ratio was no longer significantly correlated with the confidence bias in low-variance condition ($r = 0.19$, $p = 1.61e-01$). The weight accorded to the confidence report on the previous trial, called the ‘Last Confidence Multiplier’, was positively correlated with the degree of autocorrelation in the confidence reports ($r = 0.45$, $p = 7.45e-04$). (Supplementary Figure 22).

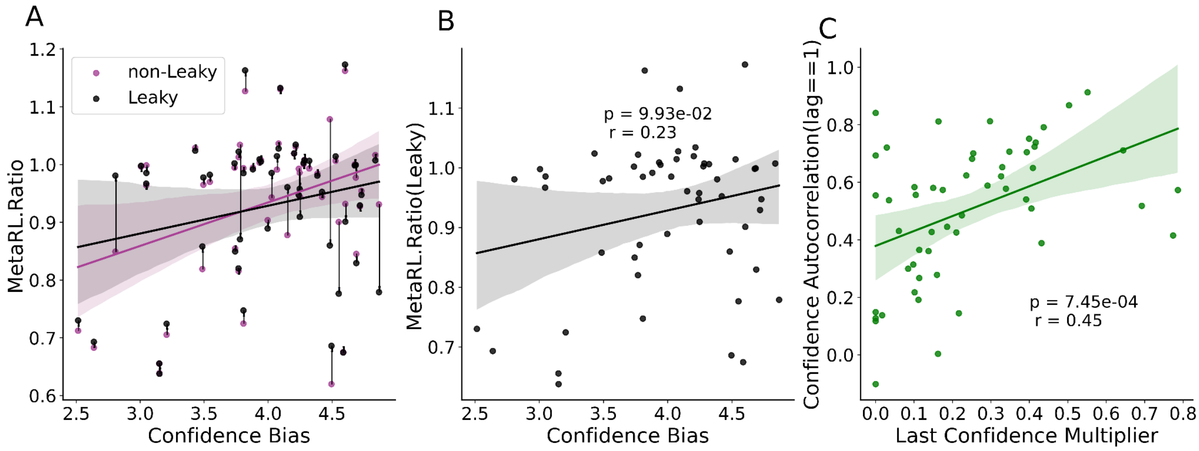


**Figure 16. Leaky model of confidence; independence from confidence bias.** We also considered an improved, ‘Leaky’ model of confidence which captures autocorrelation in confidence reports by using a weighted average of the ‘true’ confidence on the current trial and the confidence report on the previous trial. A) For some subjects the MetaRL.Ratio from leaky and non-leaky model of confidence were different from each other. B) When using this leaky model, the MetaRL.Ratio was no longer significantly correlated with the confidence bias. C) The weight accorded to the confidence report on the previous trial, called the ‘Last Confidence Multiplier’, was positively correlated with the degree of autocorrelation in the confidence reports.

However, the Leaky representation of confidence did not turn out to offer a universal solution that works across all models, as we reported in lines 355-361 of the “Result” section:

“Following the same approach as in our model-free analysis of confidence, we incorporated a leaky confidence term Lebreton et al., (2019) into the confidence representation of the Backward model. Although the leaky representation of confidence provided a better fit to empirical choices and confidence ratings compared to the basic Backward model ($M = .66$ vs. $.68$, $W = 33.5$, $p = 1.56 \times 10^{-9}$), it still failed to make the MetaRL.Ratio independent of confidence bias in this case ($r = .41$, $p = 2.12 \times 10^{-3}$).”

and discussed in lines 395-399 of the “Discussion” section:

“By applying a leaky representation of confidence (Fleming et al., 2016; Lebreton et al., 2019), which more closely matched empirical confidence reports, the MetaRL.Ratio became independent of confidence bias within the model-free (MF) reinforcement learning framework—but not in the Mixture model. Thus, the leaky confidence representation did not prove to be a universal solution across all models. It would also be interesting to explore whether the dependency of MetaRL.Ratio on confidence bias could be mitigated using different scaling methods. Investigating alternative scaling methods or distance metrics is an important next step. Notably, Shekhar and Rahnev (2021) reported a relationship between metacognitive efficiency and confidence bias, where higher or lower empirical metacognitive efficiency corresponded to higher or lower confidence bias, respectively. ”

In summary, based on the task and data, it will be interesting to explore even more accurate models of choice and confidence that might ultimately capture better the desired characteristics of a measure of metacognitive efficiency.

4. The authors framed their approach analogously to meta-SDT. Yet, meta-SDT primarily concerns a posteriori data analysis, conducted while correct or incorrect responses are sorted out. In contrast, the proposed method seems to be conducted while trial-by-trial response correctness to be unknown, aligning more with a process model perspective (similar discussions can be found in Guggenmos, 2022, eLife). I would appreciate hearing the authors' insights on this matter.

This is an interesting question. Meta-SDT does have a sort of process model for confidence reporting - otherwise, there couldn’t be a likelihood for fitting. However, in essence, we are straddling these two sets of ideas. We do share with Guggenmos the need for a process model that governs the generation of confidence. However, we share with meta-SDT the fact that what we use this process model for

is to work out what choices the confidence rater would be capable of making - and thereby judge the rater according to its possible performance on the primary task. We could certainly use a more sophisticated process model of confidence than the ones we currently describe - including the proposal of Guggenmos.

It is appealing to think about whether there would be a more model-agnostic method for extracting what the rater knows about the stimulus, based on some mutual information calculation, for instance.

We also referred to Guggenmos, 2022 at line 351 of  the “Discussion” section of our paperThanks again for your beneficial question!
