# Supplementary material for: Metacognitive efficiency in learned value-based choice
Source: PLoS Comput Biol. 2026 Mar 31;22(3):e1014108. doi: 10.1371/journal.pcbi.1014108 (PMC13102309; doi:10.1371/journal.pcbi.1014108)
Supplement: Attachment — Submitted filename: Response to Reviewers-Jan28-2026.pdf [file pcbi.1014108.s005.pdf]

Thank you very much for the editor's review of our responses and for Reviewer 1's valuable comments. They were helpful in preparing a clearer and more polished draft. We would also like to thank Reviewer 2 for accepting our responses and confirming the suitability of our manuscript. In addition, during our revisions we conducted further analyses to clarify our results, which we describe after addressing the reviewers' comments below.

118: Throughout my reading I was confused by what definition you had in mind for "confidence bias". After reading it I searched and found this parenthetic, and I think that's the definition you are using. Given the parameters that re-scale confidence, it's a wonder that anything about scaled mean confidence is all that meaningful. The scale parameters are constrained by the fits of the various models to the empirical confidence values and, in turn, are used to generate model confidence values. So, I'm not sure what it means when their averages don't match (a more or less skewed distribution over trials?). In any case, please highlight this as your definition of confidence bias.

We removed the parentheses obscuring the definition of 'confidence bias' to make it clearer at lines 119-122 of the main draft:

"Appropriately, the MetaRL.Ratio was also unaffected by task difficulty. Furthermore, given our method of matching the model's confidence ratings to empirical ones, it showed reduced dependence on confidence bias, which we quantified using the empirical average confidence (Ais et al. (2016))."

It is correct that the models' confidences were mapped to empirical confidences through scaling parameters. For both the Forward and Backward models, we identified scaling parameters that minimized the quadratic distance between the scaled confidences and the empirical ones. However, in the Backward model, the confidences were obtained by fitting the model to the empirical confidences, while in the Forward model, they were simulated based on parameters derived from fitting the Forward model to the empirical choices. After simulating the confidences from the Forward model, we estimated the scaling parameters that minimized the quadratic distance to the empirical confidences. Therefore, as expected, the Backward model's confidences were a better fit to the empirical confidences than those from the Forward model. In fact, the average confidence of the Forward model was higher than that of the Backward model. Part of the discrepancy of the mean confidence comes because of a bias variance trade-off.

168: "Figures 23 to 27" is a mismatch to the Supplement I have.

Thank you and sorry, We updated the text accordingly:

"Figures 7 to 11"

180: Here's the first use of the "confidence bias" term, so it needs to be preceded by your working definition.

In addition to updating the line 11, we updated the lines below, which are 186-187 in the updated draft:

“There was no significant difference in confidence bias—quantified using the empirical average confidence—between the (suitably scaled) Backward model and the empirical ratings (M = 3.99 vs. 3.98, W = 890, p = .205), or between the Backward and Forward model (M = 3.99 vs. 3.99, W = 691, p = .660).”

189: Again, these seem like splitting hairs, as these differences are tiny compared to the range.

Yes, we agree with your point. Since we compare model confidence with empirical confidence in terms of *average confidence*, it is reasonable that we do not observe a substantial advantage of the Backward model over the Forward model in this respect. The more important measure is, as noted in lines 206-207, the mean squared difference between trial-by-trial model and empirical confidences.

“Conversely, the Backward model predicted confidence more proficiently, with a lower quadratic distance between its and the empirical values than for the Forward model (M = .74 vs .78, W = 1, p = 1.769e-10), for all of the subjects the confidence distance between Forward and Backward models were positive (M = .033, sd = .056)”

213: additions -> addition
Figure 3 legend, next to last line: estimations -> estimates

Thank you, we updated the text accordingly, the line 213 from the previous draft is at line 222 of the current draft.

269: Another significant difference that seems insignificantly tiny

Table 1: Please get rid of scientific notation for all four of these p-values

We updated the numbers in the table and also in the text accordingly.

325-326: grammar mess-up

You are correct, we updated the line 344-345 in the updated draft as:

“We have so far only used model-free(MF) reinforcement learning (RL) methods.“

330: Latex Equation-reference messup "??"

We have updated all equation references, including the one you mentioned on line 341 of the draft.

Equations 18 to 26.

332: "was weaker" -> "was less well supported" or some such

Thank you for this suggestion, we updated the draft at line 352 accordingly.

335-336: "However, it did not fit better than". Well, it did fit better (the average AIC order is the same), just not "significantly" based on your t-test of BIC values (which is kind of a weird thing to do, even though people often do it).

Thank you very much for this helpful suggestion: we updated the text accordingly at lines 354-362:

"By contrast, the fit of the Mixture model was better than that of MF RL using AIC to penalize complexity (AIC = 193.26 vs. 224.23, W = 93, p_{Bonf}= 6.72e-08). The Mixture model also fit better based on BIC, but this difference was not significant (BIC = 213.22 vs. 232.22, W = 93, p_{Bonf} = 1.00). In addition, the Mixture model provided a better fit to participants' choices than MB RL according to both measures. (AIC = 193.26 vs. 295.44, W = 1, p_{Bonf} = 5.16e-10; BIC = 213.22 vs. 303.42, W = 5, p_{Bonf} = 6.459e-10) (see Supplementary Figure 7). Although the Mixture model's performance was marginally further from empirical' performance than MB RL (Supplementary Fig. 8), overall it was the best-fitting model of empirical' choices."

339: "Supplementary Figure 23" doesn't match my copy of the supplement

Thank you and sorry, We updated the text at line 360 accordingly:

"Supplementary Figure 7"

339-341: "were significantly closer to empirical performance" is followed by two tests that compare models to each other, not to the empirical performance. It's the next sentence that has tests that compare to empirical performance.

Thank you for your consideration. The full sentence in question was:

"The performance of the MB and Mixture models was significantly closer to empirical performance than MF (MB vs. MF: 0.87 vs. 0.76, W = 0, p = 1.626e-10; Mixture vs. MF: 0.85 vs. 0.76, W = 0, p = 1.626e-10)."

In this context, we were comparing the models to one another in terms of how closely they matched the empirical performance. As you correctly noted, the subsequent sentence reports statistical tests that directly compare each model's performance to the empirical performance. To improve clarity and make the paragraph easier to follow, we have revised this passage and replaced it with the following sentences (lines 358–360):

"Although the Mixture model's performance was marginally further from empirical' performance than MB RL (Supplementary Fig. 8), overall it was the best-fitting model of empirical' choices."

And we moved the mentioned statistics to caption of the Supplementary Fig. 8:

The performance of MB RL and Mixture models was significantly closer to participants' performance than MF RL (MB RL vs. MF RL: 0.87 vs. 0.76, W= 0, p_{Bonf} = 6.504e-10; Mixture vs. MF RL: 0.85 vs. 0.76, W = 0, p_{Bonf} = 6.504e-10). However, while the Mixture model's performance was significantly different from empirical performance (Mean = 0.85 vs. 0.86, W = 312, p_{Bonf} = 8.4e-04), MB performance was not significantly different from empirical performance (Mean = 0.87 vs. 0.86, W = 686, p_{Bonf} = 1.00). P-values were Bonferroni-corrected across the four tests reported in this paragraph; adjusted p-values are reported.

349: "was fitted" is kind of awkward phrasing

Thank you, we have updated all other instances in the text to use 'fit' as well.

352: "Supplementary Figure 26" is another mismatch

Thank you and sorry, we updated the text at lines 378-379 accordingly:

"Supplementary Figure 10"

449: les -> less

Thank you, we updated the text at line 489 accordingly.

458/462: It's weird to have "a study" connected to two different citations.

Thank you, we removed the second reference at lines 498/502.

513-514: This is a chi-squared goodness of fit to 50-50 where a reject means they were NOT guessing and should NOT be excluded. I asked for this text last time, but now see that you might as well have done an exact binomial test or the normal approximation for testing a proportion (I suppose the chi-squared is identical to that).

Thank you very much for your suggestions, which have improved the clarity of our draft. You are correct — there were some mistakes in our report. Apolgies for this - we have updated the draft at lines 562-565 accordingly:

"Participants whose performance did not exceed the chi-squared critical value of 3.841 at 1 degree of freedom (p >= .05) — that is, whose choices did not differ significantly from chance (50/50) — were excluded from further analysis."

Eq. 6: You haven't said here how you map empirical confidence (ranging from 1 to 5) to [0,1] for this equation. Eq. 12 DOES do this scaling explicitly.

In the model, we handled this differently: we fitted the L and H parameters within the bounds [1, 5], with the constraint that L< H. However, this should be equivalent to rescaling the empirical confidence values to the [0, 1] range. In the supplementary material, we report that L and H lie within the [1, 5] range.

We updated the text before and after Eq. 6 accordingly:

"... using parameters L and H, both in the range [0,5] and subject to L< H"

"In the above equation, Conf^{Fs}_t(L,H) and Conf^s_t are both in the range [1,5]."

Eq. 18: The final term P(d | \theta) as stated should probably be the probability of a duration of d or greater. Alternatively, the initial P() should include d before the conditional.

Thank you. We revised the equation accordingly to include d in the joint probability explicitly, before the conditioning, since d represents the total duration of the current state. The corrected formulation at Equation 18 now reads:

$$
\begin{aligned}
&P(S_{t-h+1:t} = i,\, r_{1:t-1},\, h, d \mid a_{1:t-1},\, \theta) = \\
&\quad P(r_{t-h+1:t-1} \mid S_{t-h+1:t-1} = i,\, a_{t-h+1:t-1},\, \theta) \\
&\quad P(S_{t-h} = j,\, r_{1:t-h} \mid a_{1:t-h},\, \theta)\, P(h \mid d)\, P(d \mid \theta),
\end{aligned}
$$

This correction clarifies that d is explicitly part of the joint distribution, making the probabilistic formulation consistent with the model description.

546: You should define all the elements of the HMM parameters rather than assuming the reader knows what each one is. I figured most of them out from later equations. I never figured out what "n" represents.

Regarding this point, we went through the section line by line and added further explanations wherever possible. In particular, we clarified the definition and role of "n" in lines 598–609:

Duration Modeling. We assume that a latent state persists for a duration d (with $d \in \{1, \ldots, D\}$, and in our implementation $D = 70$, as the maximum number of consecutive identical actions observed across subjects and conditions was 68). The duration d is modeled using a truncated negative binomial distribution:

$$P(d|D,\theta) = \frac{\binom{d+n-1}{d} p^n (1-p)^d}{\sum_{d=1}^{D} \binom{d+n-1}{d} p^n (1-p)^d}. \tag{22}$$

The negative binomial distribution, $NB(n,p)$, can be interpreted as the distribution over the number of heads observed before a fixed number of tails occur, under repeated Bernoulli trials with tail probability p. Thus, the duration d corresponds to the number of heads observed before n tails are generated. The geometric distribution is a special case of the negative binomial distribution with $n = 1$. The mean of truncated $NB(n,p)$ represented the model estimation of reversal trials, $\bar{t}_{\text{rev}}$. For each subject, the parameters n and p were fitted using a linear grid search: p was varied linearly from 0.01 to 0.99 in steps of approximately 0.06, and n from 1 to 100 in steps of 6.

Eq. 21 and line 553: It seems to me that if you want to know P(d|\theta), you are asking about a sequence that is, e.g., state 1, then state 2 for d trials, then state 1. If you condition on the first state, then the negative binomial you want is for n=1, so that n is not really a parameter.

Thank you for raising this point. In our implementation, the parameter n does **not** represent the literal number of reversals or success events; rather, it serves as the shape parameter of the negative binomial duration distribution. This is the standard parameterization used in Hidden Semi-Markov Models (e.g., Yu, 2010), where n and p together determine the mean and dispersion of the state-duration distribution. Following your comment, we have added further explanation to the manuscript, lines 598–602, as highlighted above.

Supplement: page/para/line

1/2/6: "our 56 empirical sequences". This is the first I've learned of a fixed set of sequences. One per subject? Where does this number come from?

We thank the reviewer for noting this ambiguity. The "empirical sequences" refer to the behavioral data from 54 participants (we think that the reviewer might have a typo in their comment about 56 rather than 54), each providing one sequence of 400 trials. We have clarified this in the revised text at 1/2/5 as follows:

"We simulated the Forward model to generate primary choices and confidence ratings using randomly selected task conditions from the empirical datasets of our 54 participants…"

Figure 4 legend: has a grammar mess-up (I tripped on "was a better model")

"A) The simple two-parameter forward model (1), used throughout most of the text, fits the behavioral data less well than the more complex four-parameter forward model (2). B) Even so, the performance of forward model (2) remained lower than the empirical performance (i.e., participants' performance). The dots in the plots above indicate the corresponding parameter estimates for each subject."

Figure 8 legend: "models" is missing an apostrophe somewhere

Thank you. We updated the draft accordingly.

"The performance of MB RL and Mixture models was significantly closer to participants' performance than MF RL (MB RL vs. MF RL: 0.87 vs. 0.76, W = 0, p_{Bonf} = 6.504e-10; Mixture vs. MF RL: 0.85 vs. 0.76, W = 0, p_{Bonf} = 6.504e-10). However, while the Mixture model's performance was significantly different from empirical performance (Mean = 0.85 vs. 0.86, W = 312, p_{Bonf} = 8.4e-04), MB performance was not significantly different from empirical performance (Mean = 0.87 vs. 0.86, W = 686, p_{Bonf} = 1.00). P-values were Bonferroni-corrected across the four tests reported in this paragraph; adjusted p-values are reported."

Figure 21B has "confidence bias" on the y-axis, so again a place that should follow a clear definition of how you compute confidence bias (as I say above)

We updated the caption of Figure 21B in the previous Supplementary Materials (now Figure 25B in the current Supplementary Materials) as follows:

"The confidence-bias of the Backward model, quantified as empirical average confidence, was not significantly different from that of the Forward model or empirical data, while there was a significant difference between the confidence-bias of the Forward model and empirical data."

Reviewer #2: The authors have successfully addressed all of my comments, and I would recommend the manuscript for publication.

Thank you so much again for your helpful comments on the previous version.

—---

Thank you for noting the code and data availability. We have now included direct links to both the code repository and the dataset repository in the manuscript, in the "Code and data availability" statement (lines 766–767).

"Our code is publicly available at github.com/Ershadmanesh/meta-meassure. The data are accessible via github.com/kdesende/Two-armed-bandit-confidence."

—--

Further analyses to clarify our results:

Update 1: In the previous submission, we did not report the parameters n and p for the MB RL model and for the Mixture Forward and Mixture Backward models, nor did we compare these parameters across models. We have now added these comparisons and found additional evidence supporting the Mixture models over the MB RL model. These results are described in the revised draft (lines 363–373):

> In both the MB and Mixture models, participants' prior beliefs about when reversals occur were modeled as a negative binomial distribution over block duration, summarized by its mean, $\bar{t}_{\mathrm{rev}}$, (Equations 22, Materials and Methods; see Supplementary Figure 19;20). Intuitively, larger values of the mean correspond to expecting reversals to be less frequent (i.e., longer stable blocks). We found that the absolute difference between $\bar{t}^{Fs}_{\mathrm{rev,Mix}}$ and the mean block length in the experiment (20 trials) was significantly smaller than the difference between $\bar{t}^{Fs}_{\mathrm{rev,MB}}$ and the experimental mean block length (Mean = 0.935 vs. 8.44 , W = 0, p = 1.191e-10). Thus, the Mixture model's inferred reversal times were closer to the task's true block length than those inferred by the MB model. Since the Mixture model also best fit participants' choices, this result suggests that participants were well calibrated to the task structure, reversal times.

We also added the following lines to the Discussion section (lines 421–424):

"Moreover, the Mixture model also offered a better description of participants' beliefs about block length (reversal times) than the MB formulation, suggesting that a combination of model-free and model-based computations may better capture human behaviour in this task."

And Supplementary Figures 19–21:

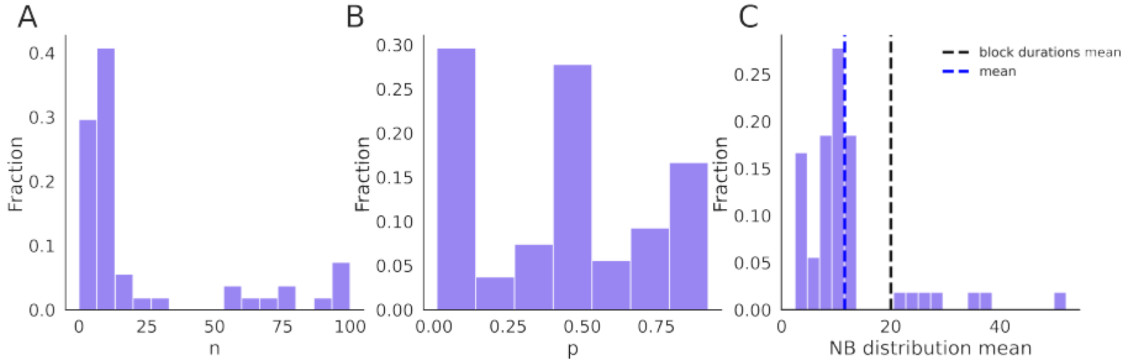

Fig S19. Parameters of the Forward MB model. A & B) Fitted n and p parameters of the Negative Binomial distribution for the Forward MB model. C)The corresponding truncated Negative Binomial distribution (capped at 70 trials). The vertical blue dashed line indicates the mean of this distribution, representing the model's reversal trials. In contrast, the vertical black dashed line indicates the mean reversal trial across all subjects and blocks in the empirical data.

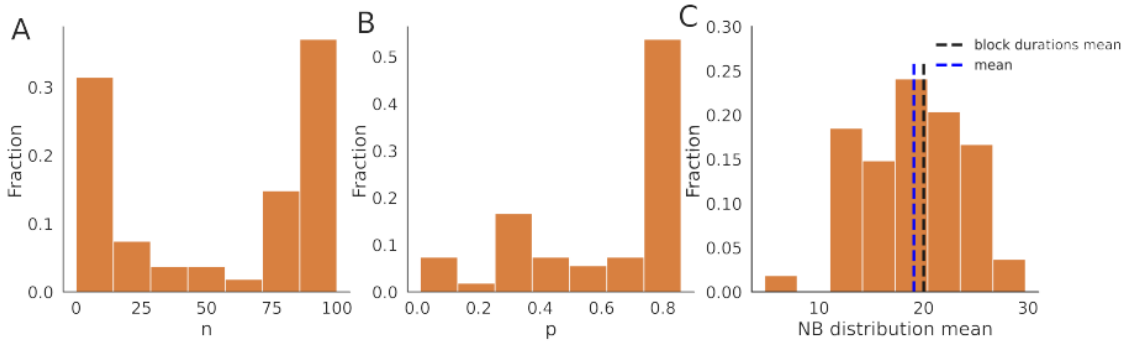

Fig S20. Parameters of the Forward Mixture model.

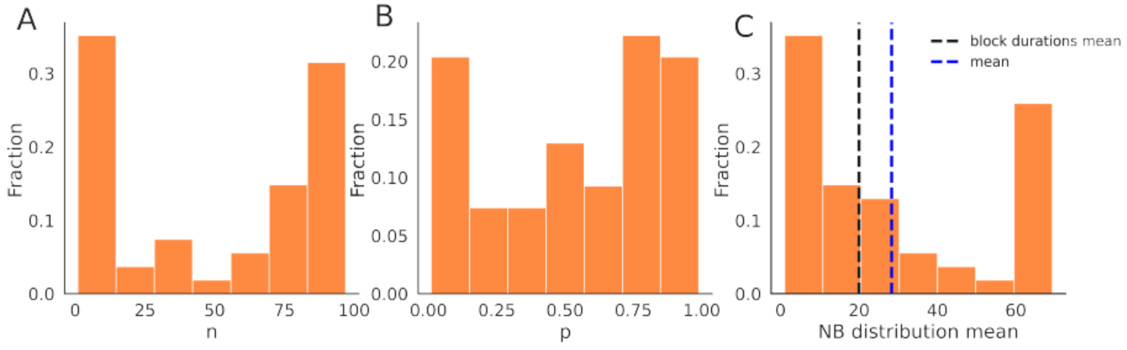

Fig S21. Parameters of the Backward Mixture model.

Update 2: In the Discussion, we noted that the learning rate in the Backward model was lower than in the Forward model and suggested that this might relate to the autocorrelation of confidence ratings. However, we did not report whether introducing the leaky version of the

Backward model increases the learning rate relative to the Backward model. To address this, we added the following text to the Results section (lines 285–292):

As we reported earlier, the learning rate of the Backward model was lower than that of the Forward model and lower learning rate of the Backward model was associated with higher auto-correlation of confidence-rating. As expected, the learning rate of the Leaky Backward model was higher than that of the Backward model (Mean = .71 vs .66, W = 98, p = 9.868e-06) while lower than the Forward model (Mean = .71 vs .94, W = 49, p = 4.836e-07) (Supplementary Figure 17). We refer to Supplementary Figure 12 for the correlation between the Leaky Backward learning rate and the autocorrelation of confidence ratings, as well as the comparison with the Backward model.

And also to the Discussion section, lines 452-460:

The learning-rate of the Backward model was significantly lower than that of the Forward model, a result we attributed to the auto-correlation in empirical confidence-ratings originally observed by (Rahnev et al., 2015; Fleming et al., 2016) and replicated here. Indeed, lower Backward learning-rates were associated with higher auto-correlation (a characteristic that a more sophisticated Backward model could eliminate; see Supplementary Figure 12). Consistently, the learning rate of the Leaky Backward model was higher than that of the Backward model while remaining lower than that of the Forward model (Supplementary Figure 17). In addition, in the low-variance condition, the Leaky Backward learning rate was not associated with the autocorrelation of confidence ratings, whereas in the high-variance condition it remained negatively associated with confidence autocorrelation (Supplementary Figure 12). This indicates that the leaky representation of confidence reduces the dependence of the learning rate on confidence autocorrelation, at least in the low-variance condition. Further work is needed to improve the confidence representation in both task conditions.

In addition, we added Supplementary Figure 17 and extended Supplementary Figure 12 to include the corresponding results for the Leaky Backward model.

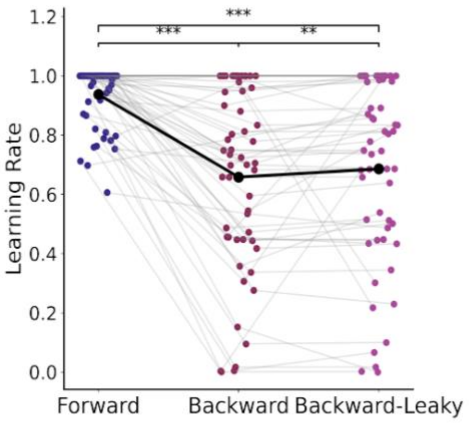

Fig S17. Comparison of learning rates between Forward, Backward, and Leaky Backward models. The Backward model had a lower learning rate than the Forward model, and the Leaky Backward model had a higher learning rate than the Backward model while remaining lower than the Forward model.

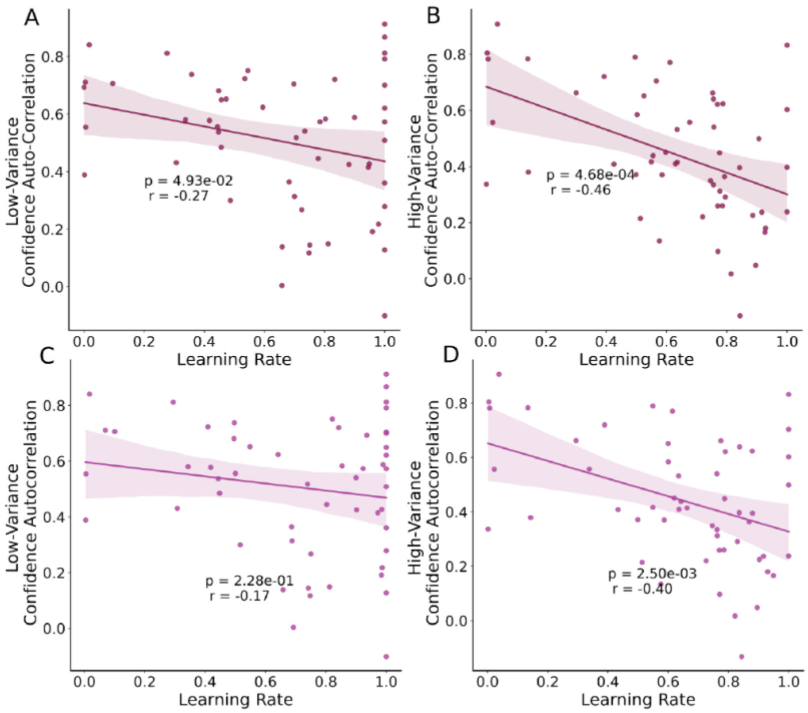

Fig S12. Backward learning-rate and auto-correlation in confidence-rates. A&B) Task conditions are shown in order (low-variance, then high-variance). In the Backward model, learning rate was negatively correlated with the autocorrelation of confidence ratings, indicating that lower learning rates were associated with more autocorrelated confidence ratings: low-variance ($r = -0.27$, $p = 4.93e-02$) and high-variance ($r = -0.46$, $p = 4.68e-04$). C&D) The same analysis for the Leaky Backward model. In the low-variance condition, the correlation was not significant ($r = -0.17$, $p = 2.28e-01$), whereas in the high-variance condition, lower learning rates were associated with higher autocorrelation ($r = -0.40$, $p = 2.50e-03$). Each dot represents one participant. The strength of the correlation did not differ significantly between the Backward and Leaky Backward models in either condition (low-variance: $Z = 0.53$, $p = .59$, high-variance: $Z = 0.37$, $p = 0.71$).

Update 3: In the previous draft, we used the terms “accuracy” and “performance” interchangeably to refer to the proportion of trials in which participants chose the best option. We also used “performance” when stating that one model “outperformed” another based on how well it fit the empirical data, which could be confusing.

In the revised manuscript and Supplementary Materials, we no longer use the term "accuracy." Instead, we use "performance" exclusively to mean the proportion of trials in which the best option was chosen, and we apply this definition consistently to both empirical and model choices (i.e., "empirical/model performance" refers to the same measure). When comparing models in terms of how well they match the data, we now use "better/worse fit" rather than "outperformed." We highlighted all updated words in both the main draft and the supplementary materials.
